# Transfer learning for a foundational chemistry model†

**DOI:** 10.1039/d3sc04928k

**Published:** 2023-11-24

**Authors:** Emma King-Smith

**Affiliations:** a Cavendish Laboratory, University of Cambridge Cambridge UK esk34@cam.ac.uk

## Abstract

Data-driven chemistry has garnered much interest concurrent with improvements in hardware and the development of new machine learning models. However, obtaining sufficiently large, accurate datasets of a desired chemical outcome for data-driven chemistry remains a challenge. The community has made significant efforts to democratize and curate available information for more facile machine learning applications, but the limiting factor is usually the laborious nature of generating large-scale data. Transfer learning has been noted in certain applications to alleviate some of the data burden, but this protocol is typically carried out on a case-by-case basis, with the transfer learning task expertly chosen to fit the finetuning. Herein, I develop a machine learning framework capable of accurate chemistry-relevant prediction amid general sources of low data. First, a chemical “foundational model” is trained using a dataset of ∼1 million experimental organic crystal structures. A task specific module is then stacked atop this foundational model and subjected to finetuning. This approach achieves state-of-the-art performance on a diverse set of tasks: toxicity prediction, yield prediction, and odor prediction.

## Abstract 2

Harnessing knowledge from crystal structures yields a model that can predict a variety of chemistry-relevant outcomes.

## Graphical abstract

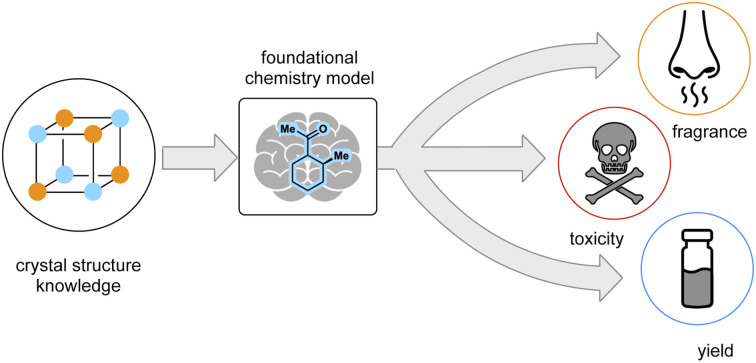

## Introduction

The implementation of computerized algorithms into organic chemistry has had a rich history, with early emphasis centered around deriving linear relationships from observed results.^1^ By the 1970s, synthetic chemists had turned their attention to utilizing more complex functions to model more abstract observations. In 1977, Corey *et al.* published the first recognized retrosynthetic analyzer, LHASA (Logics and Heuristics Applied to Synthetic Analysis) which featured hand coded expert rules resulting in over 30 000 lines of FORTRAN code.^2^ With the turn of the century, improvements in computational hardware and the development of new computer learning algorithms, including machine learning (ML), have seeded new avenues for algorithm-based predictive chemistry.^3,4^ ML is the process of taking complex inputs, abstracting their relevant features through non-linear equations, and correlating those features to a given output. Despite its simplistic framework, variations upon this theme have yielded advances in numerous areas including fundamental molecular property prediction (*e.g.*, quantum chemical, ADMET), reaction property prediction (*e.g.*, regioselectivity, yield), and generative modeling.^5–7^ With these more powerful algorithms come higher data requirements. Where the first data-driven chemistry models may have necessitated a few experimental results, ML often demands tens of thousands of data points. Purely computational datasets from density functional theory (DFT) or semi-empirical methods have been generated and utilized in ML for prediction of quantum chemical properties (*e.g.*, HOMO–LUMO gaps, dipole moments)^8^ and in molecular scaffold generation.^9^ Benefits of using these datasets include larger sizes and less noise present within each observation. Whilst experimental data is inherently noisier, more expensive, and often more laborious to generate than computational data, it presents a more holistic representation of a chemical system, even if we do not fully understand the intricacies present within that system. It is therefore important for the community to find ways to incorporate these smaller, experimental datasets as a key feature into ML tools.

**Fig. 1:**
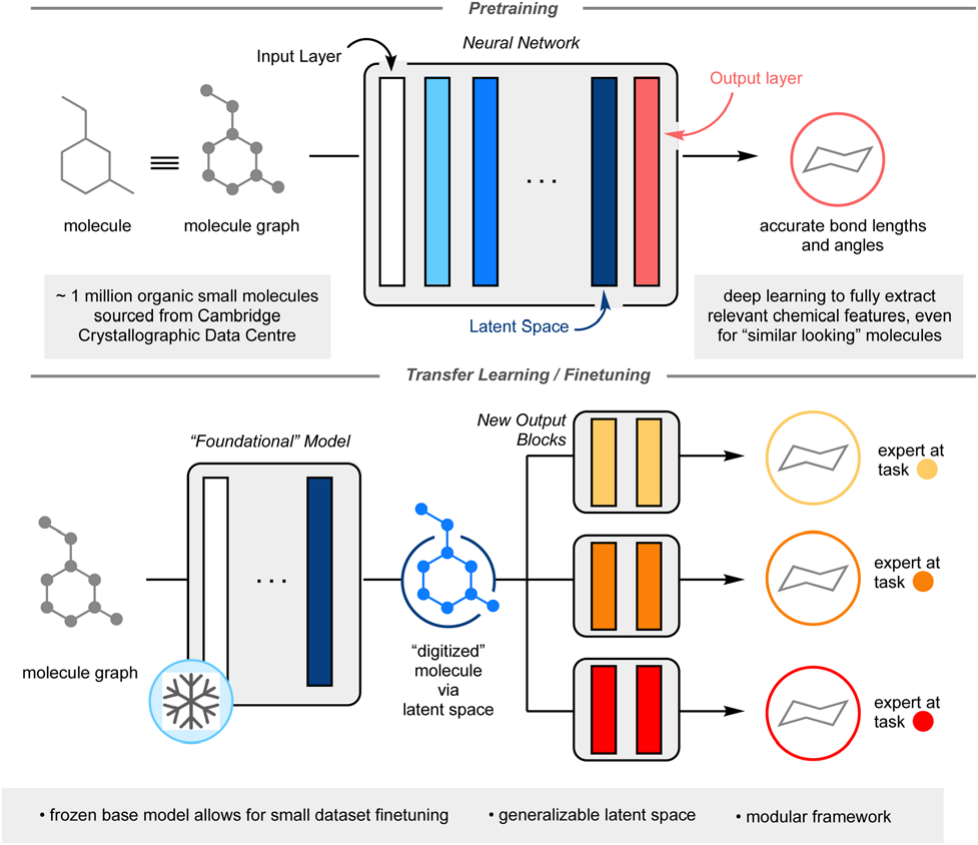
Graphical overview of the framework. Top panel illustrates the pretraining process and the structure of deep learning neural networks. The bottom panel shows how the top large model (foundational model) can be used for new chemistry-relevant predictions *via* the foundational model's latent space.

One method that has seen potential towards the utilization of smaller datasets in deep ML is transfer learning.^10^ In this process, a model is first trained on a large dataset. The target prediction of the first task (pretraining task) does not need to be directly related to the desired final task (finetuning task), however, the initial knowledge gained from pretraining must have some relevancy to the finetuning. In a neural network, each non-linear function is referred to as a layer; each layer in the neural network is responsible for extracting relevant chemical features for a given task. The first layer is defined as the input layer, the final layer as the output layer, and for this manuscript, the penultimate layer is defined as the latent space (Fig. 1). One may imagine the latent space as a complete digitization of a molecule, whereby each molecular feature has been assigned a series of numbers. The key to effective transfer learning hinges around this latent space, whereby each molecule's chemically relevant features are so well characterized that the substitution of one output layer for another output layer results in accurate prediction of a different chemical property (see ESI: A non-expert's guide to transfer learning (CliffsNotes version) on p. S3 for further explanation of transfer learning†). In essence, the model transfers the knowledge it learnt from pretraining to finetuning. To date, transfer learning in data-driven chemistry has been applied on a case-by-case basis, where pretraining tasks are expertly chosen for specific finetunings to minimize domain mismatch.^10^ This limits the ML possibilities for smaller datasets.

Herein, I report the development of a general chemistry-centric foundational model utilizing transfer learning, capitalizing upon the molecular featurization from the resultant latent space. Rather than concocting a molecular representation through manual descriptor selection like in traditional QSAR, an underlying model, dubbed the “foundational model” is utilized to generate the molecular representation, from which further training can be carried out to predict any endpoint properties of choice in a modular fashion, re-using (transferring) knowledge acquired in the first step. The goal of the foundational model is to ensure that enough relevant chemical information is present in the molecular representation (See ESI: A non-expert's guide to transfer learning (CliffsNotes version) on p. S3 for further explanation of transfer learning†). This work's proposed foundational model is trained to accurately predict molecular bond lengths and angles. Approximately 1 million experimentally validated organic crystal structures from the Cambridge Crystallographic Data Centre (CCDC) was used to train this foundational model.^11^ It was hypothesized that an ML model capable of predicting accurate crystal structure information would contain a latent space useful in the prediction of many other chemical outcomes. It was envisioned that the size and scope of the CCDC would allow for a deep neural network approach, capable of inferring nuanced interactions between atoms and/or motifs from a given molecule. Importantly, as only the final output block of the neural network would be undergoing training, this would yield a modular, flexible framework capable of reliable prediction even on limited training data. The modularity and accuracy of this approach on three chemistry-related prediction tasks: toxicity prediction, yield prediction, and olfaction prediction are reported, showcasing a performance improvement over other documented ML techniques and, when applicable, comparison to other deep learning models (Fig. 1).

## Results & discussion

The first task was to generate a foundational model whose latent space could digitize a molecule for subsequent downstream finetuning. As the CCDC dataset is centered around crystal structure data, whose focus is on the geometry of the molecule(s), I opted for a graph-based model. Graph-based models represent molecules as mathematical graphs, interpreting atoms as abstract objects and bonds as indicators of a relationship between two atoms.^8,12,13^ The specific graph neural network utilized was a message passing neural network (MPNN), a flavor of graph convolutional neural network which have noted success in a variety of chemistry prediction tasks.^8,13–17^ Briefly, an MPNN deduces the local chemical environment for each atom within the molecule, preserving the symmetry of chemically identical atoms. The training set for this initial task was comprised of carbon-containing crystal structures in the CCDC. Molecules which contained “rare” atoms (atoms that were represented fewer than 100 times in the dataset) were excluded, which still yielded a broad atomic scope (Fig. S2†). Additionally, structures whose bonding pattern was ambiguous and conformational polymorphs with a significant difference in their conformations were also removed. A large message passing neural network was trained to accept 2D-data of a molecular structure and predict an atomic coordinate proxy of each atom within the molecule. Atomic coordinates are not unique to a molecule or conformation (a molecule rotated through space is the same molecule, but its 3D coordinates will have changed), thus the model predicted the through-space distances of an atom to its nearest neighbors and the corresponding bond angles formed. It was discovered that such an MPNN could accurately predict the bond lengths and angles of unseen molecules (scaffold split) (Table S1†). Thus, the investigation into the transferability of the foundational model's latent space commenced. It should be noted that crystal structure data has been used to predict solid form and crystal structure based properties but has seen limited application in transfer learning to more tangential tasks.^18,19^ Given the potential foundational chemistry knowledge that could be extracted from the CCDC dataset, it was hypothesized a transfer learning approach from CCDC data would traverse an unexplored gap within the data-driven chemistry literature.

With the trained foundational model in hand, the final output layer was discarded and replaced with a new, untrained feedforward neural network. This new network was shallow (2 linear layers) and small (*e.g.*, ∼32k parameters for toxicity and olfactive prediction) to allow for rapid and facile training towards other chemistry-centric tasks. The pretrained layers, the bulk of this system, are “frozen”: no additional training is performed upon them. For the following examples, little to no further hyperparameter optimization was conducted to highlight the ease of translation from foundational model to finetuned model (Fig. 1).

To validate the foundational model's applicability as a “springboard” for other chemistry tasks, three datasets were sourced and used for finetuning that covered a broad range of structure-to-function prediction tasks: acute toxicity, Suzuki and Buchwald–Hartwig yield regression, and odor classification. These tasks are of interest to areas within data-driven chemistry, given their utility to drug discovery and development, chemical synthesis, and perfume production and have open-source datasets available for modelling and facile benchmarking.^20–22^ Additionally, these chemical tasks have little overlap with one another; high toxicity of a molecule has little bearing on its cross coupling yields. Thus, the extent and breadth of the foundational model's molecular representation can be interrogated.

### Toxicity

The connection between molecular structure and toxicity has been well documented and has been subject to excellent QSAR modelling.^23,24^ Thus, it was believed that toxicity prediction was a natural first exploration for the modular framework. For this proof-of-concept, the regression prediction of therapeutic toxicities (LD_50_) from basic, 2D structural information of a given small molecule drug was attempted. Data was sourced from the Therapeutics Data Commons (TDC), a repository of drug relevant data for both small molecules and biologics providing useful benchmarks for *in silico* drug design.^25^ For this task, the dataset of interest was the Acute Toxicity dataset consisting of 7358 small molecule pharmaceutics. This dataset had a built-in scaffold split which was used for their benchmarking. A scaffold split partitions the dataset so that the test set has consists of molecules excluded from the training set. This is considered to be a more challenging target than a random split, whereby randomly chosen molecules are assigned to the test set. Specifically, it is believed that there is less data leakage when employing a scaffold split than a random split.^25^ Additional investigation of the TDC training (5170 molecules) and testing (1447 molecules) sets revealed a high level of similarity in the distribution of LD_50_ values and molecular sizes. However, the TDC test set had notably fewer molecules with LD_50_ values in the 500–1000 mg kg^−1^ range (Fig. S3†). For this toxicity prediction task and all subsequent tasks, an unseen molecules test set policy (scaffold split) was employed. TDC leaderboards from time of writing indicate a top model, Oloren ChemEngine, with an accuracy of 0.552 mean absolute error (MAE) (eqn (1)), indicating that on the test set, Oloren ChemEngine predicted, on average, within ±0.552 units of the true experimental value. For reference, a perfect model would have an MAE = 0. Oloren ChemEngine is an open-source flexible system, which removes much of the code writing for task-specific prediction, instead choosing the best molecular, occasionally proprietary, featurization and model for the user's data.^20^1
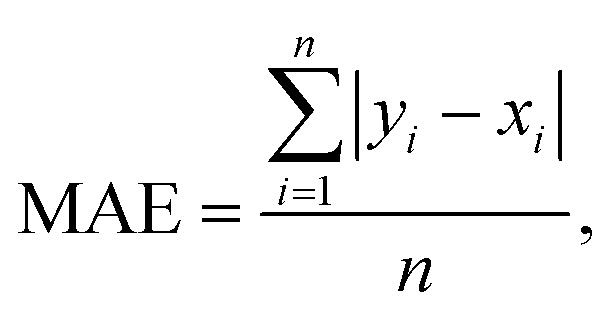
where *y*_*i*_ are the true values and *x*_*i*_ are the predicted values.

**Fig. 2:**
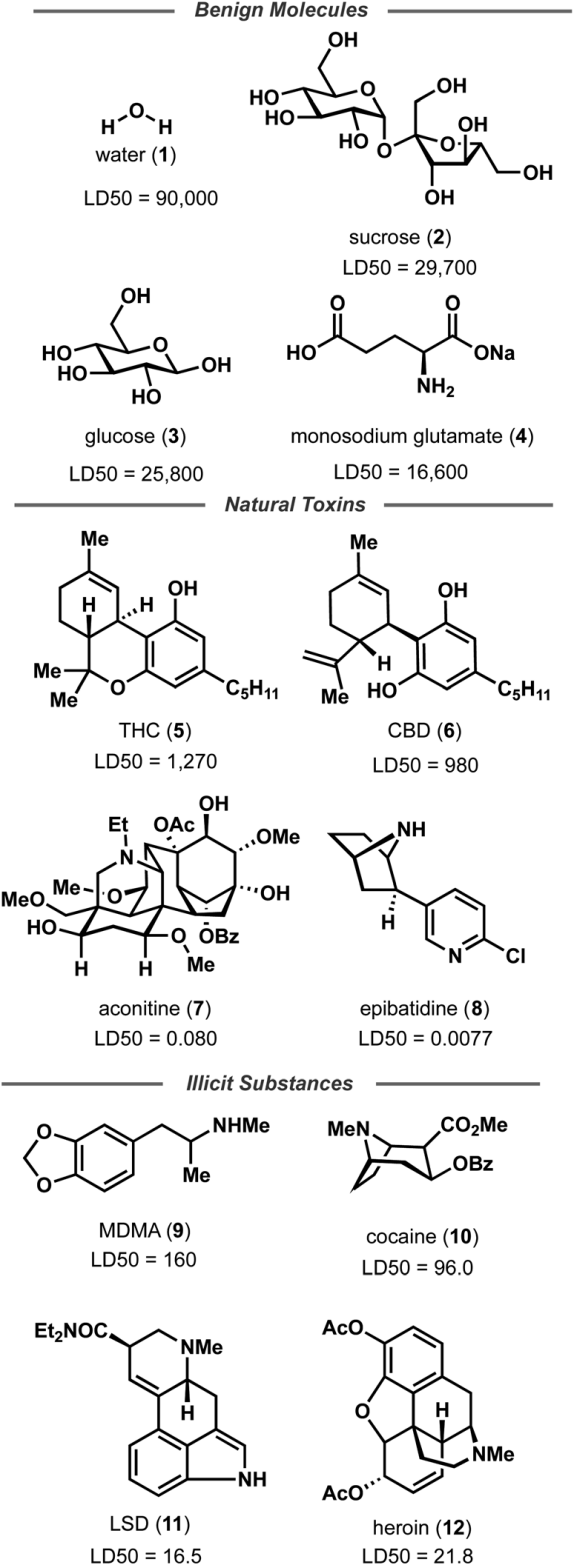
Non-therapeutic small molecules to challenge the toxicity prediction models.

**Fig. 3:**
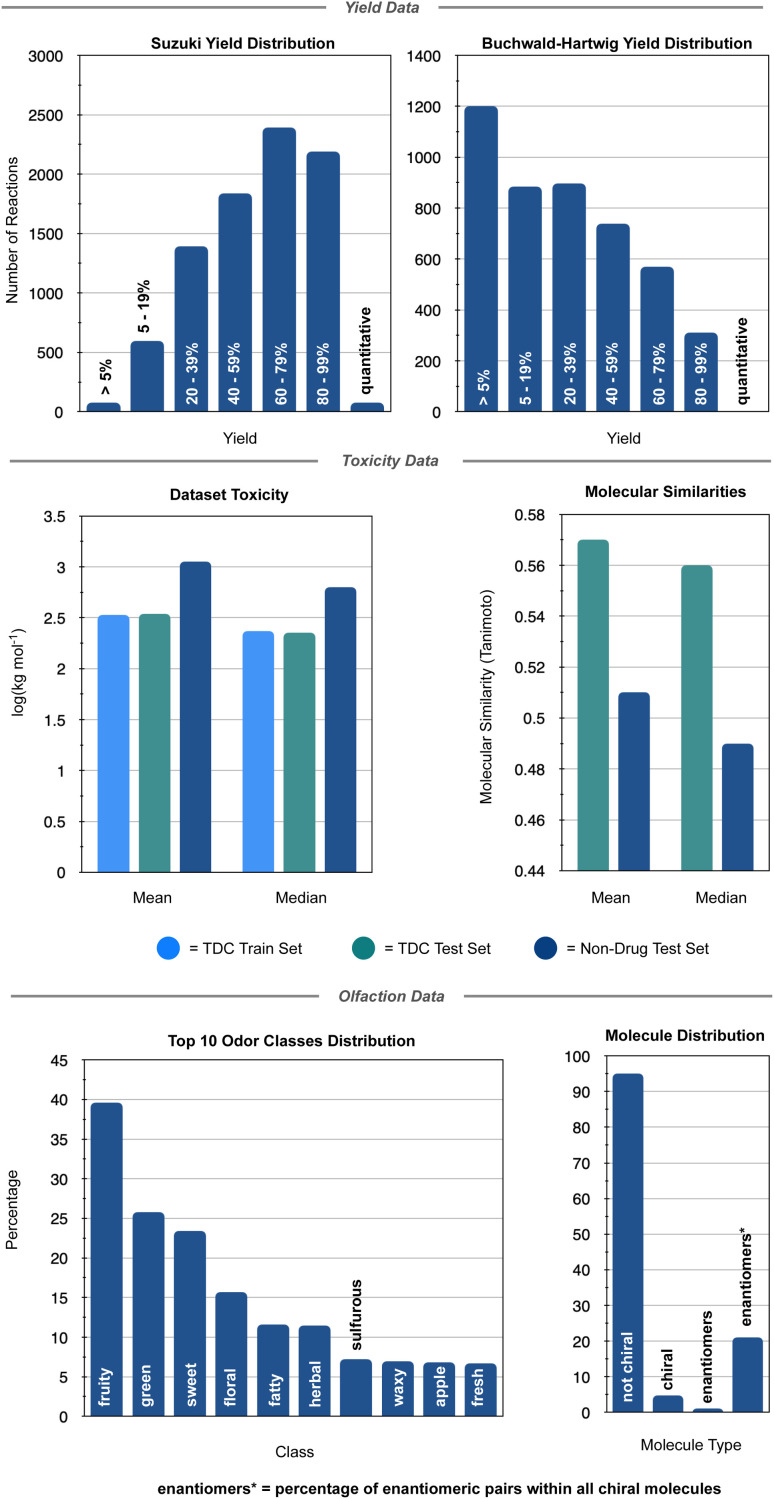
Dataset breakdowns for the three chemistry prediction tasks. Top panel showcases the yield distributions for the Suzuki and Buchwald–Hartwig datasets. Middle panel shows the mean and median toxicities and compound similarities of the training and testing LD_50_ datasets. Bottom panel highlights the diversity of olfactive classes and the limited occurrence of chiral molecules in the Pyrfume dataset for machine olfaction.

Random forest, Gaussian process, and Adaboost models, all of which have noted good performance on molecule property prediction, were chosen as baseline models.^26–28^ Superior performance of Oloren ChemEngine (lower MAE) on TDC's test set against these three baseline models was noted (Table 1). The model pretrained on CCDC data, Crystal-Tox, was modestly more accurate than Oloren ChemEngine and significantly more accurate than the three baselines with an average MAE of 0.52 over 5 initializations. As the hypothesis was that pretraining from crystal structure data would yield a model with more chemical knowledge than models without pretraining, each model and baseline was challenged to predict the toxicities of non-therapeutic molecules across a greater range of chemical space. To this end, 12 molecules were chosen: 4 benign molecules (water, sucrose, glucose, and monosodium glutamate), with benign defined as an LD_50_ value greater than 1500 mg kg^−1^, 4 natural toxins (THC, CBD, aconitine, and epibatidine), and 4 illicit substances (MDMA, cocaine, LSD, and heroin) (Fig. 2). This new test set was comprised of molecules which had not been included in any of the training iterations and represented a different distribution of chemical toxicity. Mean and median toxicity values (units = log(kg mol^−1^)) for this non-therapeutics test set were 3.05 and 2.79, notably higher than the mean and median toxicity values for the training set of 2.53 and 2.36 (Fig. 3). TDC test set mean and median toxicity values were near identical to the TDC training set's values. Additionally, the minimum toxicity values were lower in this new test set than in the training set, −0.70 compared to −0.34. Compared to the TDC training and testing sets, which were comprised of solely pharmaceutical compounds, this test set had a substantially different distribution of LD_50_ values. The proportion of molecules with an LD_50_ value of 10 000 mg kg^−1^ or higher was 33%, compared to 0.5% (TDC training set) and 3.7% (TDC testing set). Approximately 50% of TDC training set molecules had LD_50_ values in the 500–4000 mg kg^−1^ range. Only 17% of compounds in the non-therapeutics test set had LD_50_ values between 500–4000 mg kg^−1^ (Fig. S3†). These new molecules were also, on average, structurally less similar to the training data than the TDC's test set (Fig. 3 and S3†). In summary, these new molecules represented a greater slice of chemical toxicity space than had been previously trained or tested upon. Unsurprisingly, baseline models' performance upon testing on the aforementioned compounds dropped significantly to 1.54 (random forest), 1.86 (Gaussian process), and 1.73 (Adaboost) MAE. Oloren ChemEngine still outperformed baselines with a mean MAE of 1.46 with Crystal-Tox showing again a modest improvement over all 4 models with an MAE of 1.38 (Table 2). Crystal-Tox was most accurate at compounds in the mid-range toxicity scale, with the most benign (water) and most toxic (epibatidine) having far less extreme predicted toxicity values than reality (Table S2†). Interestingly, both Oloren ChemEngine and Crystal-Tox correctly identified the higher toxicity of aconitine, also known by its common names “wolfsbane” and “monkshood”. The high accuracy of Crystal-Tox was primarily due to its better understanding of non-toxic substances, which were often ranked as ∼0.5 units more toxic by Oloren ChemEngine's best model (Table S2†). Crystal-Tox's outperformance over baselines and Oloren ChemEngine models highlighted the promising nature of this framework towards toxicity prediction of pharmaceutical compounds and toxic, non-pharmaceutical compounds.

**Table 1:** Evaluation of baseline models, the Therapeutic Data Commons' (TDC) top model, Oloren ChemEngine, and this work's toxicity model, Crystal-Tox, on the TDC's test set and the test set comprised of non-therapeutic molecules. Metric used was mean absolute error (MAE) metric. Best error has been bolded

Model	TDC test set (MAE)	Non-drug test set (MAE)
Random forest	0.62 ± 0.002	1.59 ± 0.02
Gaussian process	0.73 ± 0.002	1.86 ± 0.002
Adaboost	0.71 ± 0.002	1.77 ± 0.002
Oloren ChemEngine	0.55 ± 0.009	1.46 ± 0.006
Crystal-Tox	**0.52 ± 0.007**	**1.38 ± 0.02**

**Table 2:** Evaluation of baseline models, Yield-BERT, and this work's yield prediction model, Crystal-Yield, on Suzuki and Buchwald–Hartwig yield predictions. Metric used was mean absolute error (MAE) metric. Best error has been bolded

Model	Suzuki yields MAE (unseen boronic acids)^a^	Suzuki yields MAE (unseen halides)^a^	Buchwald–Hartwig (unseen halides) MAE^b^	Buchwald–Hartwig (unseen bases) MAE^b^	Buchwald–Hartwig (unseen ligands) MAE^b^	Buchwald–Hartwig (unseen additives) MAE^b^
Random forest	19.5 ± 0.03	19.5 ± 0.03	25.2 ± 2.0	28.1 ± 4.1	28.5 ± 0.6	30.4 ± 1.5
Gaussian process^c^	—	—	26.3 ± 1.9	30.0 ± 2.8	33.0 ± 2.4	29.3 ± 1.2
Adaboost	21.6 ± 0.1	21.5 ± 0.1	24.7 ± 2.6	25.5 ± 2.9	27.9 ± 0.7	27.6 ± 0.5
Yield-BERT	21.9 ± 0.06	22.0 ± 0.03	24.7 ± 2.1	24.3 ± 1.6	24.3 ± 1.4	24.1 ± 0.7
GraphRXN	40.0 ± 3.0	37.8 ± 2.7	25.2 ± 7.0	17.9 ± 4.6	13.8 ± 1.7	17.5 ± 1.8
Crystal-Yield	**18.4 ± 0.03**	**18.5 ± 0.2**	**21.3 ± 3.3**	**13.4 ± 0.3**	**11.7 ± 2.2** d	**16.2 ± 0.4**

a Most common reactant was used as the test split. Standard error determined from 3 initializations.

b Each molecule was used in tested against in *k*-fold validation (unseen molecules in each fold). Standard error determined from each fold. Test performance prediction on each fold can be found in Table S3.

c Poor predictive performance on Suzuki prediction thus used as a baseline machine learning model for Buchwald–Hartwig data only.

d Crystal-Tox whose final output layer has been increased from ∼130k parameters to ∼1 million parameters. For reference, GraphRXN had ∼2 million parameters.

### Reaction yields

Reaction yield prediction, both qualitative and quantitative, has been a rich area for machine learning in chemistry. Driven by its importance to synthetic chemists and by the relative abundance of data through patent literature curation (USPTO) and high throughput experimentation (HTE), several notable ML models have been generated with the intention of accurate yield prediction.^21,29,30^ It has been noted that a model with good yield prediction accuracy could be utilized for a number of valued fields including reagent ranking and retrosynthesis design, a challenging field often considered by synthetic chemists to showcase the “artform” of synthetic chemistry.^31^ The link between solid state molecular structure and reaction outcomes, which are typically performed in solution, is more tenuous than for toxicity, thus yield prediction is a challenge use case for foundational chemistry knowledge extracted from structural data. Two reactions which have seen enormous utility in the synthetic community are the Suzuki and Buchwald–Hartwig coupling reactions, highly versatile palladium-catalyzed carbon–X bond formation reactions. Indeed, Suzuki and Buchwald–Hartwig couplings made up nearly a third of all reactions performed in medicinal chemistry and natural product total synthesis in 2016, with its prevalence only increasing in the following years.^32^

Attention was first directed to Suzuki coupling reactions, whose data was sourced from the US patent literature (USPTO).^33,34^ Prior deep learning models have noted excellent performance, sometimes only a 5% discrepancy between experimental and predicted values.^21,29,30^ However, when a leave-molecule-out approach was taken on Buchwald–Hartwig coupling data, whereby the test molecules are unseen molecules, model performance drops precipitously. The model is therefore challenged on this style of splitting, where the top reagents/reactants are excluded from training and seen for the first time during model evaluation. This Suzuki dataset comprised of 5143 electrophiles, 1122 nucleophiles, 10 catalysts, and 90 ligands (Fig. S4†). When the most common boronic acids are removed from the training data to be used as the test dataset, a modest difference in yield and product size distribution between the training and testing sets is observed. Leaving out the most common aryl halides for model testing resulted in a more significant change between the training and testing set yield and product size distributions (Fig. S5†). These two metrics indicate a modest level of similarity between each training and testing set of unseen molecules. Both test sets were ∼10% the size of the training datasets (Fig. S6†). Similar to toxicity predictions, yield prediction baselines of random forest and Adaboost with MAE as the metric were used. Initial trials with Gaussian process regression models yielded far lower accuracies, thus they were omitted from the baseline measure. Subjecting each baseline model against the test reactions with unseen boronic acids or aryl halides yielded a modest performance of 19.5 (random forest, unseen boronic acids)/21.6 (Adaboost, unseen boronic acids) and 19.5 (random forest, unseen halides)/21.5 (Adaboost, unseen halides) average MAE. Yield-BERT, a transformer-based model which acts as a machine translation from the language of reaction SMILES to product yield, was used as an accurate ML benchmark. Yield-BERT has been reported to have excellent performance on both Suzuki and Buchwald–Hartwig yield prediction, even when trained on limited data, making it a suitable benchmark for my framework. Additionally, similar to this pretraining approach, Yield-BERT uses no precomputed data and even outperforms modeling with DFT-based chemical descriptors.^29^ A large MPNN, GraphRXN, was similarly chosen as an additional benchmark. GraphRXN had observed notable success on external and internal HTE data. Interestingly, Yield-BERT achieves similar accuracy on the dataset splits to random forest, yielding 21.9 and 22.0 MAE on unseen boronic acid and aryl halides, respectively. GraphRXN performs worse that Yield-BERT, a surprising outcome given that modeling of GraphRXN on Suzuki, albeit HTE data and not the historical, collated, literature patent data used in this interrogation, was on-par with Yield-BERT.^21^ The framework pretrained from CCDC data, dubbed Crystal-Yield, showed a modest performance increase across both the top nucleophile and electrophiles test sets, with a mean MAE of 18.4 for unseen nucleophiles and a mean MAE of 18.5 for unseen electrophiles (Table 2).

The similarity in performance of the models is likely due to the inherently noisy nature of experimental chemistry: where different chemists, different reagent lots, and different environments can cause shifts within the experimental value. By its very nature, the USPTO Suzuki dataset is comprised of reactions from multiple chemists across the country and across decades of research. Indeed, historical bias is often present in this style of collated data, which can translate to poor machine understanding.^35^ Additionally, negative data is rarely observed in this dataset, with only 0.9% of all reactions reporting a yield under 5% (Fig. 3). Prior research have noted the importance of this “negative data” in predictive modelling.^15,36^ Crystal-Yield's average MAE of ∼18% is a step forward towards accurate modeling of noisier data.

To showcase the framework's potential on more systematically sampled data, reaction yields of Buchwald–Hartwig cross couplings generated by Ahneman *et al.* were modeled.^37^ Unlike the Suzuki coupling data, reaction yields were determined solely through high throughput experimentation (HTE) from a single laboratory and 26% of the dataset consisted of “negative reactions” (Fig. 3). For this dataset, a single amine is reacted against 16 aryl halides, 3 bases, 4 ligands, and 24 additives (Fig. S4†). The test sets formed from leaving out each reaction component resulted in a marked difference in training and test set yield distributions (Fig. S7–S10†). This indicates that each test set represents a challenge. Similar to the Suzuki modelling, each test set was ∼10% the size of the training data (Fig. S6†). Prior modeling revealed that testing on unseen additives can lead to much higher error rates.^29,30^ Notably, additives are not the only critical factor in yield determination, thus, I probed the performance of Crystal-Yield when predicting on not just unseen additives, but unseen aryl halides, bases, and catalysts. Baseline models of random forest, Adaboost, and Gaussian process were used. As with each previous test set, unseen molecules (halides, bases, ligands, additives) were used. Model performance for the Buchwald–Hartwig reactions was the average of each molecules' test set performance, which consisted of several halides/bases/ligands/additives that were excluded from training. Once again, similar performance of Yield-BERT and the baseline models was observed, however, GraphRXN clearly showcased its specialization in modelling HTE data. Notably, when the base Crystal-Yield model (∼260 000 parameters) was used to predict Buchwald–Hartwig yields on unseen ligands, GraphRXN outperformed with an MAE of 13.8 compared to Crystal-Yield's MAE of 18.2. However, increasing the size of the modular output block to ∼1 million parameters, 50% the size of GraphRXN, delivered a significant improvement in Crystal-Yield's performance (MAE = 11.7). Similar good accuracy was achieved on the other splits with average MAEs of 21.3 (unseen halides), 13.4 (unseen bases), and 16.2 (unseen additives) (Tables 2 and S3†). A closer evaluation showed that even the lower scoring splits, such as testing on unseen halides, were primarily caused due to 1 or 2 poorer performing test splits, indicating especially difficult modeling of those particular molecules (Table S3†). With the exception of five splits, Crystal-Yield was the most accurate across each set of unseen molecules. This showcased the foundational model's generalizability across two highly distinct chemistry prediction tasks, even when compared to a model specifically built for HTE-derived prediction (GraphRXN).

### Fragrance

**Fig. 4:**
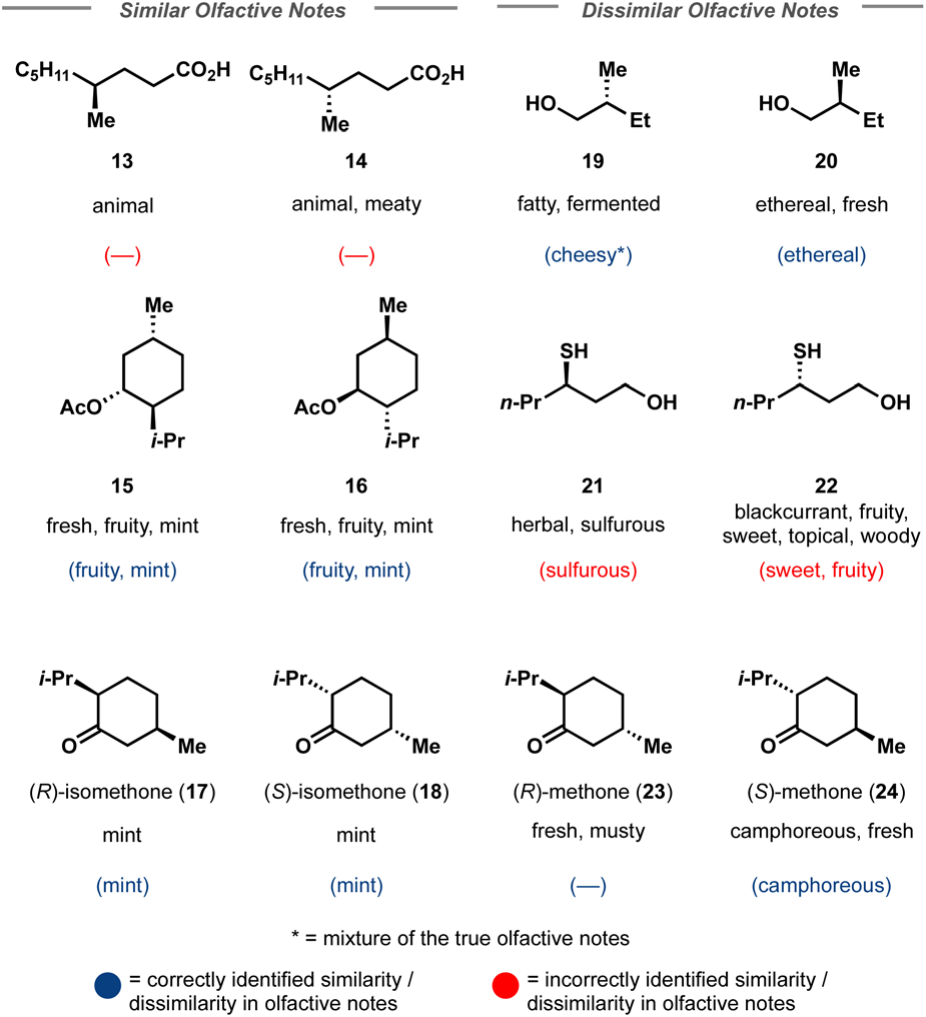
Select enantiomeric pairs used as the challenging test set for Crystal-Olfaction. Correct top 5 predictions are shown in parentheses where a horizontal line indicates that none of the top 5 most likely labels were correct. Blue predictions show that Crystal-Olfaction correctly identified that the enantiomeric pair had an identical/differing olfactive profile, even if no label was correctly predicted in the top 5. Red predictions indicate that Crystal-Olfaction determined incorrectly identified the similarity of scent profile between the enantiomeric pair.

Finally, model finetuning on molecule odor prediction was investigated. Similar to toxicity, perceived fragrance is highly dependent upon molecule structure, with most humans being able to distinguish between select enantiomers. However, odor is, by definition, perceived odor and the current mechanism by which olfactive detection and recognition occurs is still opaque.^38^ Indeed, it is well known that two individuals may interpret a molecule's fragrance differently or be anosmic to a given molecule (unable to smell the molecule). Thus, the finetuning task is not a regression, but a multilabel multiclass prediction. Multilabel refers to the fact that there are many possible odors for a molecule, and whilst there is no universal definition of fragrance classes, there are generally accepted realms: sweet *vs.* herbaceous *vs.* floral. Multiclass indicates the possibility that a molecule may have multiple possible olfactive notes; a molecule may smell sweet and buttery, not just one or the other. It can be observed from the fragrance distribution of the dataset that many molecules offer a fruity component (Fig. 3) which may itself be accompanied by a specific fruit, such as melon or blackcurrant (Fig. 4).

Whilst Lee *et al.* and Sanchez-Lengeling *et al.* have published on the potential for MPNNs in odor mapping and fragrance prediction, as of writing, they have not yet made their raw code available for benchmarking.^22,39^ As such, I used two standard multilabel, multiclass classification baselines: random forest and *K*-nearest neighbors. Previously used baselines Adaboost and Gaussian process are unable to work with multilabel, multiclass classification in their standard scikit implementation. Random forest models have been noted previously for their excellence in machine olfaction.^40^ Small molecule fragrance data was obtained from the Pyrfume project, an open-source database of pre-processed, literature-curated olfactive data.^41^ This dataset consisted of 3502 small molecules each with a corresponding label which could be any number of combinations from the 113 odor classes. Interestingly, a minority of molecules in this dataset had no detectable fragrance (Fig. S11†). A cross validation (5-fold) split was performed, where each molecule in the test split was an unseen molecule. Unlike yield and LD_50_ prediction, which were regression tasks, classification was to be performed and thus, the *F*-score classification metric was utilized. *F*-scores are typically used for binary classifications but the multiclass extension of the *F*-score has been developed. Briefly, the *F*-score for each class is computed before being averaged. The averaging can be unweighted (macro) or weighted by class size (weighted). For *F*-scores, higher values indicate better model performance. With random forest and *K*-nearest neighbor baselines, relatively low *F*-scores were observed, highlighting the challenge of predicting reasonable olfactive notes from structurally different molecules. However, my crystal structure foundational model combined with an olfactive-specific feedforward neural network, Crystal-Olfaction, observed a generous increase in both weighted and unweighted *F*-scores to 0.62 and 0.92 (Table 3). One notable challenge for all networks was the prediction of non-fragrant molecules, which were all incorrectly assigned with multiple odor labels. This may be due to a dataset bias of few non-fragrant molecules or it is possible that these molecules have an olfactive profile, but the volatility of these compounds is so low, that they are not detectable to most people. I believe this to be an interesting future direction for further exploration of this modular framework.

**Table 3:** Evaluation of baseline models and this work's fragrance prediction model, Crystal-Olfaction on compound odor test sets. Metrics used were macro *F*-scores and weighted *F*-scores. Best error has been bolded

Model	Macro *F*-score (5-fold CV)	Weighted *F*-score (5-fold CV)	Macro *F*-score (enantiomeric pairs)	Weighted *F*-score (enantiomeric pairs)
Random forest	0.19 ± 0.01	0.32 ± 0.009	0.069 ± 0.002	0.31 ± 0.003
*K*-Nearest neighbors	0.20 ± 0.002	0.33 ± 0.002	0.031 ± 0.0002	0.2 ± 0.001
Crystal-Olfaction	**0.62 ± 0.004**	**0.92 ± 0.002**	**0.58 ± 0.003**	**0.93 ± 0.002**

Finally, Crystal-Olfaction was challenged to distinguish between not just structurally dissimilar molecules, but between enantiomers, distinguished by a one-hot coded chiral tag. It is well understood that whilst their physical properties are identical, enantiomers can be perceived as two distinct scents. A classic example is that of carvone, where (*R*)-carvone is often described as spearmint and (*S*)-carvone as caraway. Thus, 11 enantiomer pairs, 5 of which have identical olfactive profiles and 6 which have distinct olfactive profiles were chosen. These enantiomeric pairs were different from the training dataset, both in terms of molecule size and in their assigned fragrance classes, of which little overlap was observed (Fig. S12†). Crystal-Olfaction and the baseline models were trained on all training data folds (3578 molecules) and tested on these 22 compounds (Fig. 4 and S13†). It was noted that all models found this new test set difficult, although Crystal-Olfaction performed significantly better than baselines (Table 3). Given the baseline models' difficulty in distinguishing odor classes on structurally dissimilar molecules, it is unsurprising that enantiomer distinctions were similarly challenging (Fig. 4). Crystal-Olfaction was more accurate at determining the correct scent labels, however, its ability to distinguish between enantiomeric olfactive profiles was more limited. Of the 11 pairs, 5 were correctly identified as having identical or different fragrance notes (Fig. S4†). Commonly, Crystal-Olfaction would predict identical odor classes for enantiomers with substantially different scents. However, some successes were observed: the differentiation between menthone enantiomers 23 and 24 and of enantiomers 19 and 20. Whilst none of the top 5 predicted labels for 19 were correct, a “fermented fatty” odor could be construed as cheesy, a label that Crystal-Olfaction deemed highly likely for 19. The challenge of this test set is highlighted in the low density (5%) of chiral molecules in the training dataset, of which only 21% of those chiral molecules were enantiomeric pairs (Fig. 3). Inclusion of additional chiral molecules into the training set may yield more discernment between enantiomer pairs.

## Conclusions

I have demonstrated a proof-of-concept for a foundational chemistry model, capable of predicting toxicity, palladium catalyzed cross coupling yields, and molecule fragrance from the 2D structures of these compounds. Key to this success was the utilization of a subset of the CCDC's dataset to generate a foundational model with enough chemical knowledge to be applicable across a range of chemistry fields. This was showcased with the modular development of three finetuned models, which outperformed baselines and other deep learning model benchmarks. The foundational model as well as the trained models are offered to the public for future exploration of toxicity, reaction outcome, and olfactive predictive modeling as well as other chemistry-relevant tasks such as structure activity relationship exploration (SAR) and reagent design.

## Data availability

The code, foundational model, trained models, and associated datasets can be accessed at our GitHub repository: https://github.com/emmaking-smith/Modular_Latent_Space.

## Author contributions

The work, in its entirety, was conceived and carried out by EKS. The manuscript was written by EKS.

## Conflicts of interest

The author declares no competing interests.

## Supplementary Material

SC-015-D3SC04928K-s001
