# Supplementary material for: Transfer learning for a foundational chemistry model
Source: Chem Sci. 2023 Nov 24;15(14):5143–51. doi: 10.1039/d3sc04928k (PMC10988575; doi:10.1039/d3sc04928k)
Supplement: SC-015-D3SC04928K-s001 [file SC-015-D3SC04928K-s001.pdf]

Electronic Supplementary Material (ESI) for Chemical Science.
This journal is © The Royal Society of Chemistry 2023

Supporting Information

Transfer Learning for a Foundational Chemistry Model

Emma King-Smith*

Correspondence to: esk34@cam.ac.uk

Table of Contents

Materials and Methods.. page S3
Code Availability.. page S3
A Non-Expert's Guide to Transfer Learning (CliffsNotes Version)............................ page S3
Table S1.. page S4
Table S2.. page S5
Table S3.. page S6
Figure S1... page S7
Figure S2... page S8
Figure S3... page S9
Figure S4... page S10
Figure S5... page S11
Figure S6... page S12
Figure S7... page S13
Figure S8... page S14
Figure S9... page S15
Figure S10... page S16
Figure S11... page S17
Figure S12... page S18
Figure S13... page S19
User's Guide.. page S20-S44

Materials and Methods:
Training and testing of the deep neural networks was performed on a server with 16-core dual threaded AMD Ryzen 95950X processors running CUDA 11.4. Dataset cleanup of the Suzuki dataset, Buchwald-Hartwig dataset, toxicity datasets, and olfaction datasets was carried out on a 2020 MacBook Air M1 (MacOS Big Sur 11.2.3) with PyCharm IDE (2021.1.1 Community Edition).

Code Availability:
The base model latent space parameters and finetuned model parameters are available on the GitHub repository (https://github.com/emmaking-smith/Modular_Latent_Space/tree/master). Cleaned, open-source datasets and all code have also been made available. Access to the Cambridge Crystallographic Data Centre's (CCDC) Cambridge Structural Database (CSD) Python API and data can be accessed by entering a private access agreement with CCDC.

A Non-Expert's Guide to Transfer Learning (CliffsNotes Version):
Transfer learning is the process whereby the information gathered from one source (pretraining dataset) is used to "jump start" the learning from a second source (finetuning dataset). Typically, the finetuning dataset is the desired prediction target. If the finetuning dataset is small and data augmentation through other sources or experimental design is not possible or cost prohibitive, transfer learning is a potential solution. It allows the user to use a bigger model with the bigger pretraining dataset, which may lend itself to better results on the finetuning dataset task (finetuning task). Transfer learning may also be thought of as a way pointing the model in the correct direction.

Practically, the transfer learning is as follows. A machine learning model is trained on the pretraining dataset to predict a pretraining task. The final layer, used to formulate the pretraining task predictions, is removed and replaced with a new layer that will be used to predict the finetuning dataset task. The previous neural network layers retain the information learned from the pretraining dataset task. From here, the user may opt to "freeze" the pretrained layers or to re-train the whole system. Freezing the layers allows for small datasets to be used with deep neural networks. Re-training the whole system may be more beneficial if both datasets are sufficiently large and sufficiently distinct from one another (Figure S1).

Model	Test Set MSE
Small MPNN	3.17
Large MPNN	2.93

Table S1: Mean Squared Error (MSE) of total loss (bond distance loss + bond angles loss) on crystal structure data for a variety of message passing neural networks (MPNNs). Test set consisted of unseen molecules.

Compound	True Toxicity (log(mol kg^{-1}))	Crystal-Tox Predicted Toxicity (log(mol kg^{-1}))	Oloren ChemEngine Predicted Toxicity (log(mol kg^{-1}))
water	–0.70	1.53	1.98
sucrose	1.06	1.01	1.48
glucose	0.84	1.25	1.77
monosodium glutamate	1.00	1.66	2.10
THC	2.39	2.88	2.53
CBD	2.51	2.62	2.41
aconitine	6.90	3.84	3.38
epibatidine	7.43	2.88	2.93
MDMA	3.08	2.59	2.55
cocaine	3.50	2.09	2.67
LSD	4.29	2.65	2.89
heroin	4.23	2.80	3.19

Table S2: Predicted and true toxicity values of each compound in the non-drug test set for the best Crystal-Tox and Oloren ChemEngine models.

Model	Split MAE			
	Halide Set 0	*Halide Set 1*	*Halide Set 2*	*Halide Set 3*
Random Forest	23.6	23.9	22.2	31.0
Gaussian Process	27.3	25.2	21.7	30.9
Adaboost	24.6	23.9	18.7	31.6
Yield-BERT	27.3	25.2	21.7	30.9
GraphRXN	**9.5**	41.6	30.9	**18.7**
Crystal-Yield	26.7	**14.8**	**16.3**	27.5
	Base 0	*Base 1*	*Base 2*	
Random Forest	32.0	32.4	19.9	
Gaussian Process	31.0	34.3	24.8	
Adaboost	27.2	29.5	19.9	
Yield-BERT	23.3	27.4	22.1	
GraphRXN	**12.8**	27.1	13.8	
Crystal-Yield	13.9	**13.0**	**13.4**	
	Ligand 0	*Ligand 1*	*Ligand 2*	*Ligand 3*
Random Forest	27.4	29.0	27.6	29.8
Gaussian Process	39.8	32.2	29.2	30.6
Adaboost	26.8	29.9	25.9	27.2
Yield-BERT	20.4	24.0	25.8	27.0
GraphRXN	**9.7**	17.6	12.7	15.2
Crystal-Yield	24.5	23.4	10.4	14.5
Crystal-Yield[a]	17.1	**12.2**	**6.5**	**10.8**
	Additive Set 0	*Additive Set 1*	*Additive Set 2*	*Additive Set 3*
Random Forest	34.0	31.3	26.7	29.4
Gaussian Process	32.7	29.0	24.5	27.9
Adaboost	29.0	27.3	26.7	27.5
Yield-BERT	25.2	22.9	22.8	25.3
GraphRXN	16.7	15.2	22.8	**15.4**
Crystal-Yield	**15.6**	**16.6**	**17.2**	15.5

Table S3: The mean absolute error (MAE) for each fold in the Buchwald-Hartwig yield prediction. For halides and additives, several were left out at a single time to allow for equal training-testing splits for all validations. Bolded entries indicate the best model for each fold. [a]Crystal-Yield with output block increased from ~260K parameters to ~1 million parameters. GraphRXN had ~2 million parameters.

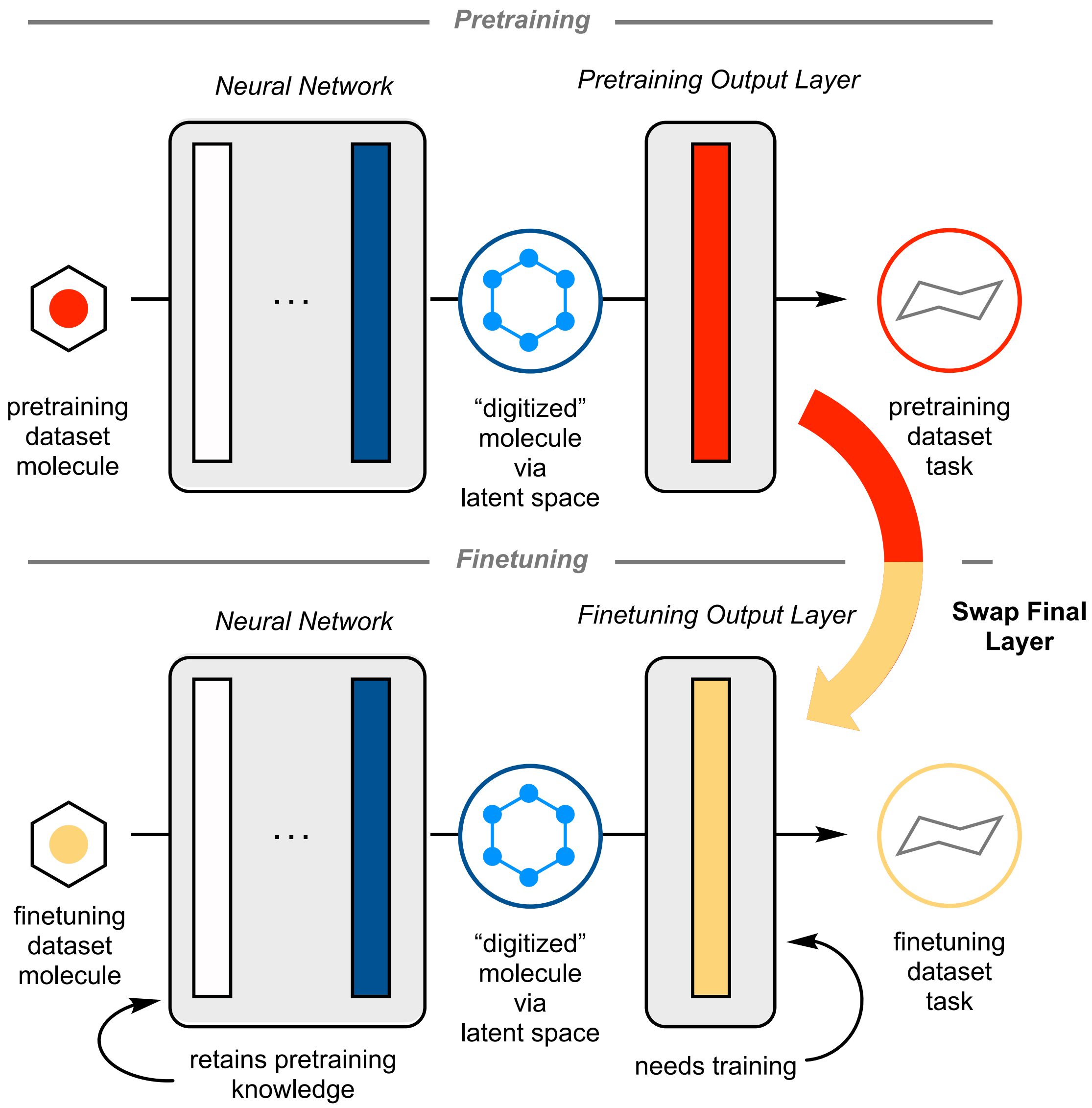

Figure S1: Graphical representation of the process of transfer learning. The neural network may be frozen (no more training occurs) if the finetuning dataset size cannot accommodate the depth of the whole system.

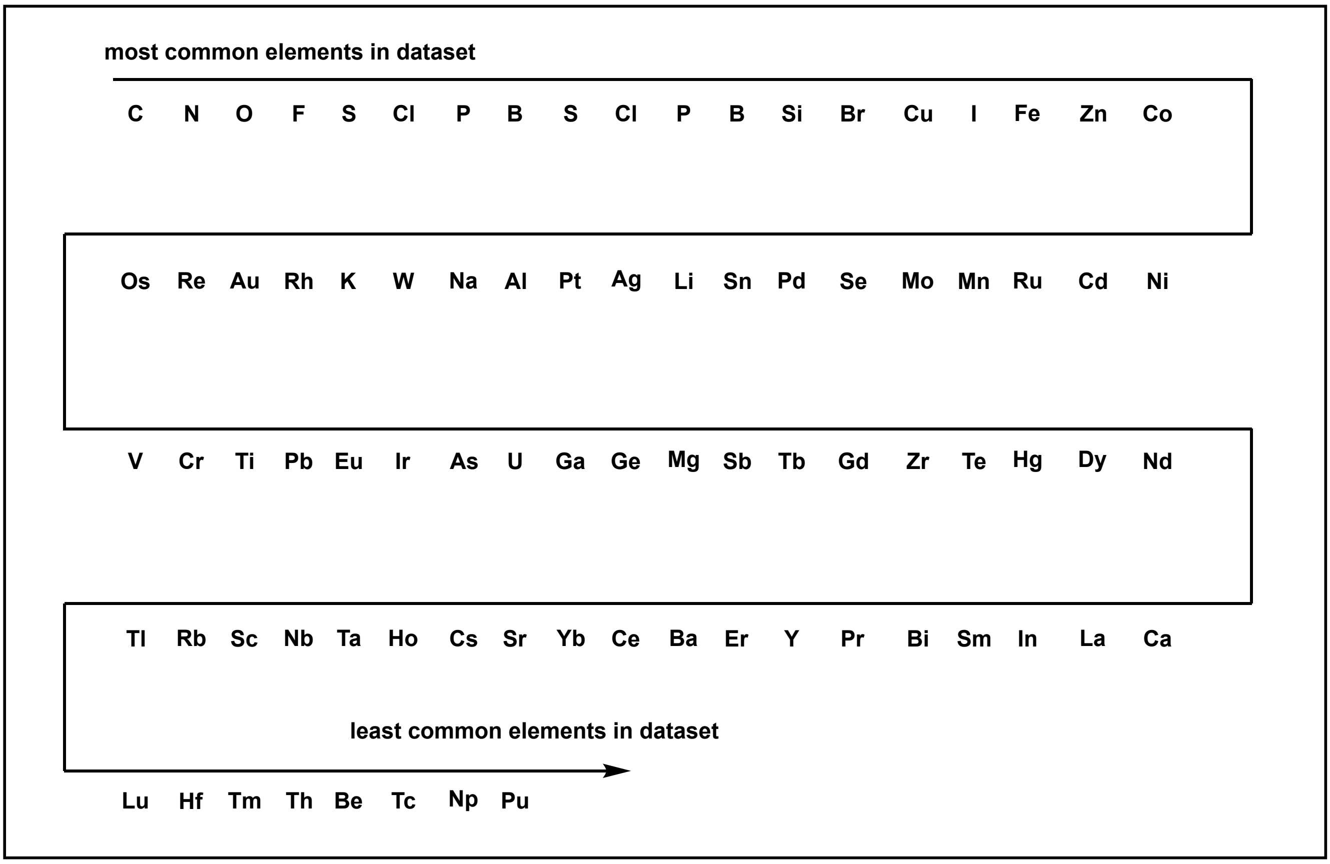

Figure S2: Ranking of elements in our cleaned CCDC dataset.

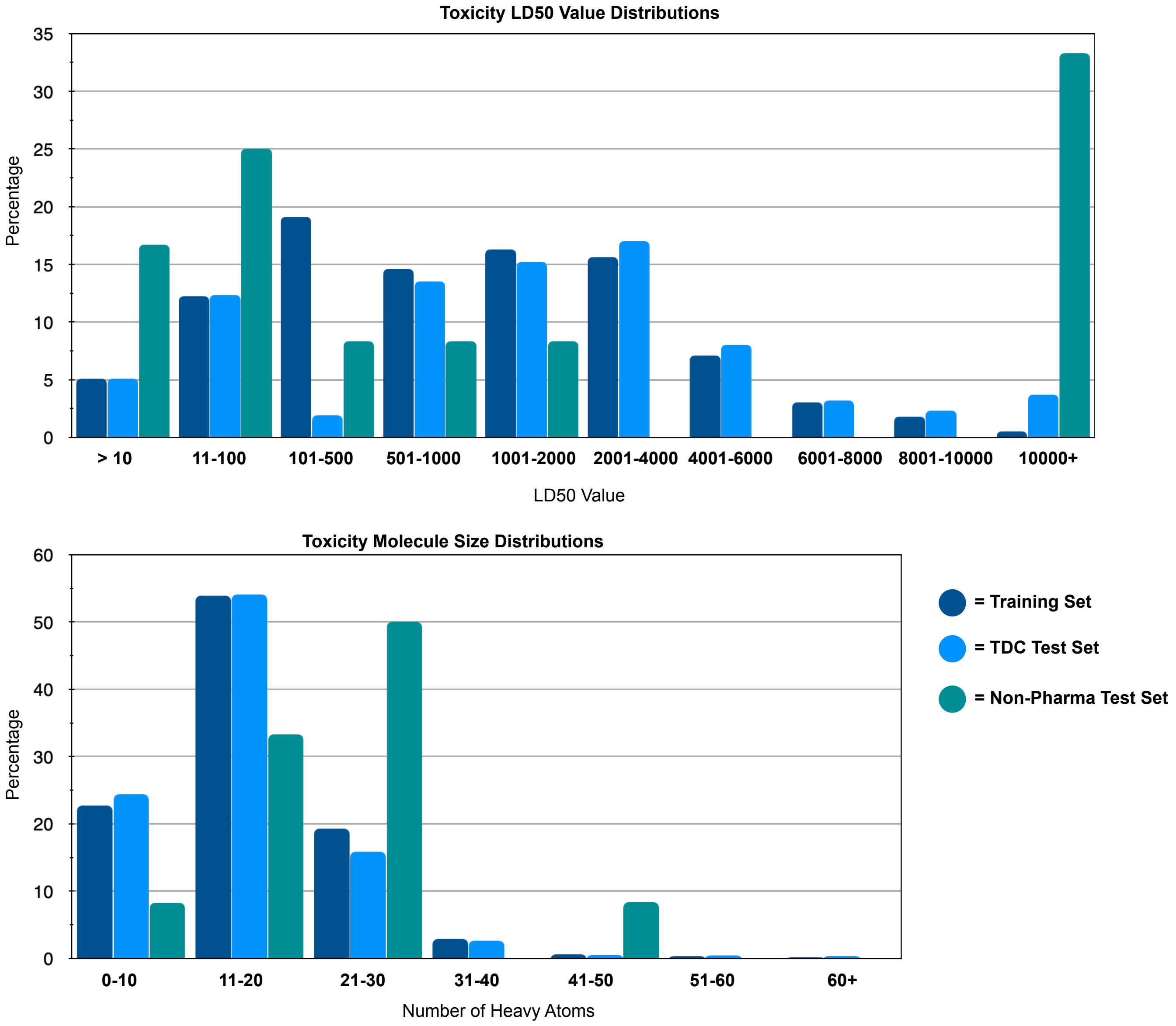

Figure S3: Distribution of LD50 values and molecular sizes for the toxicity finetuning tasks. Dark blue bars indicate training set distribution, light blue bars indicate the TDC testing set distribution, and teal bars indicate the non-pharmaceutical testing set distribution.

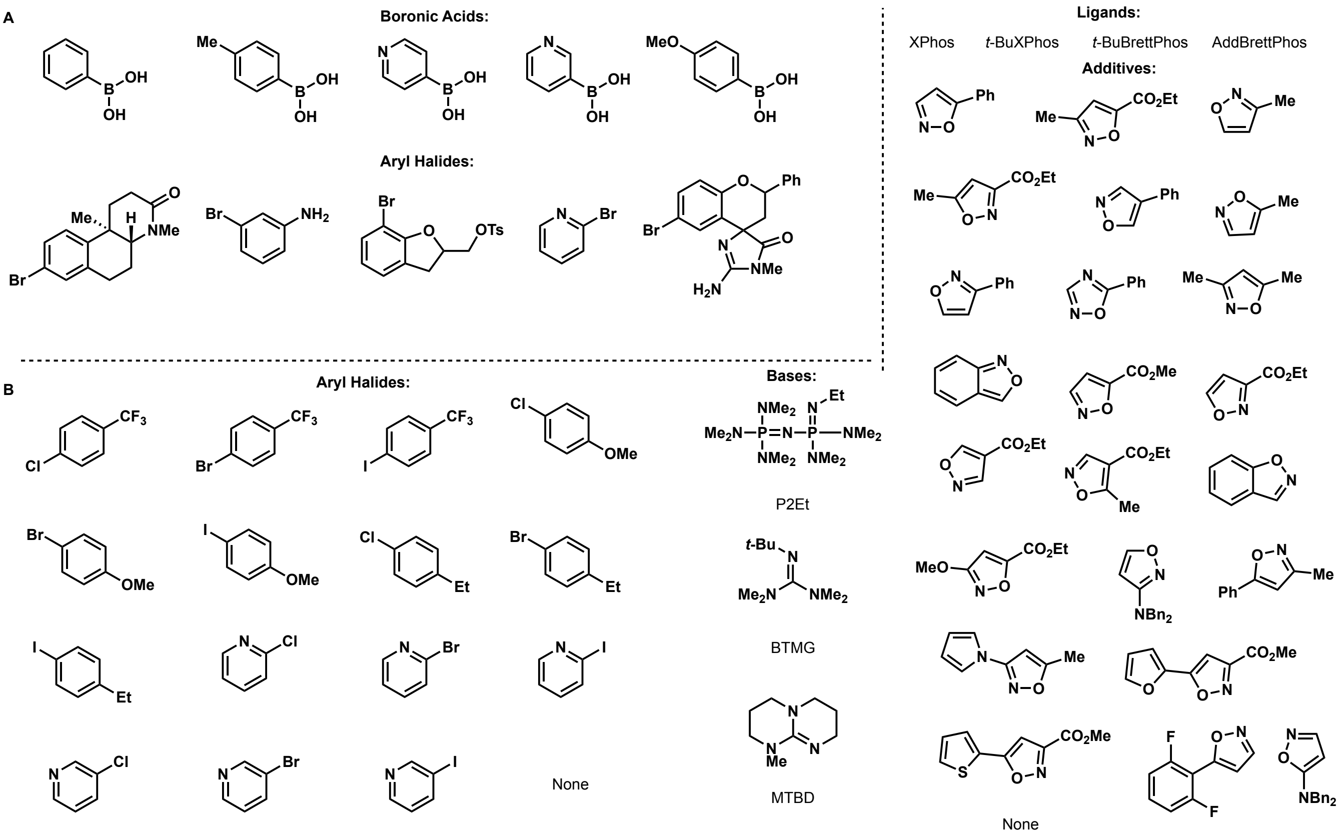

Figure S4: Representative scopes of reaction yield datasets. (**A**) Top 5 boronic acids and aryl halides in the USPTO Suzuki coupling dataset. (**B**) All halides, bases, ligands, and additives used in Buchwald-Hartwig coupling dataset.

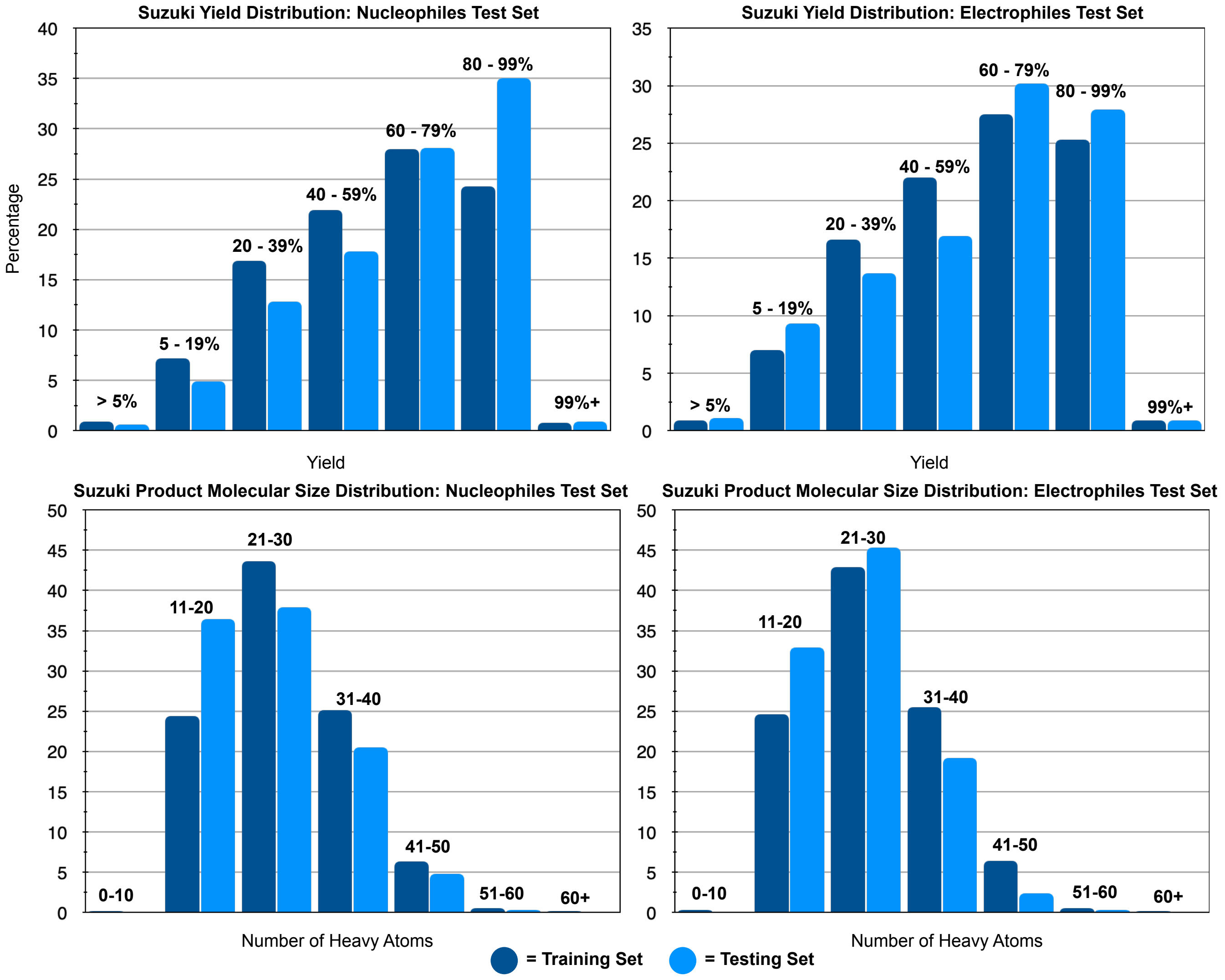

Figure S5: Suzuki test set and training set comparisons for each test set, consisting of molecules not present in the training set. Dark blue bars indicate training set distribution and light blue bars indicate testing set distribution. **Top:** Comparisons of yield distributions of training and test sets. **Bottom:** Comparisons of product molecule size distributions of training and testing sets.

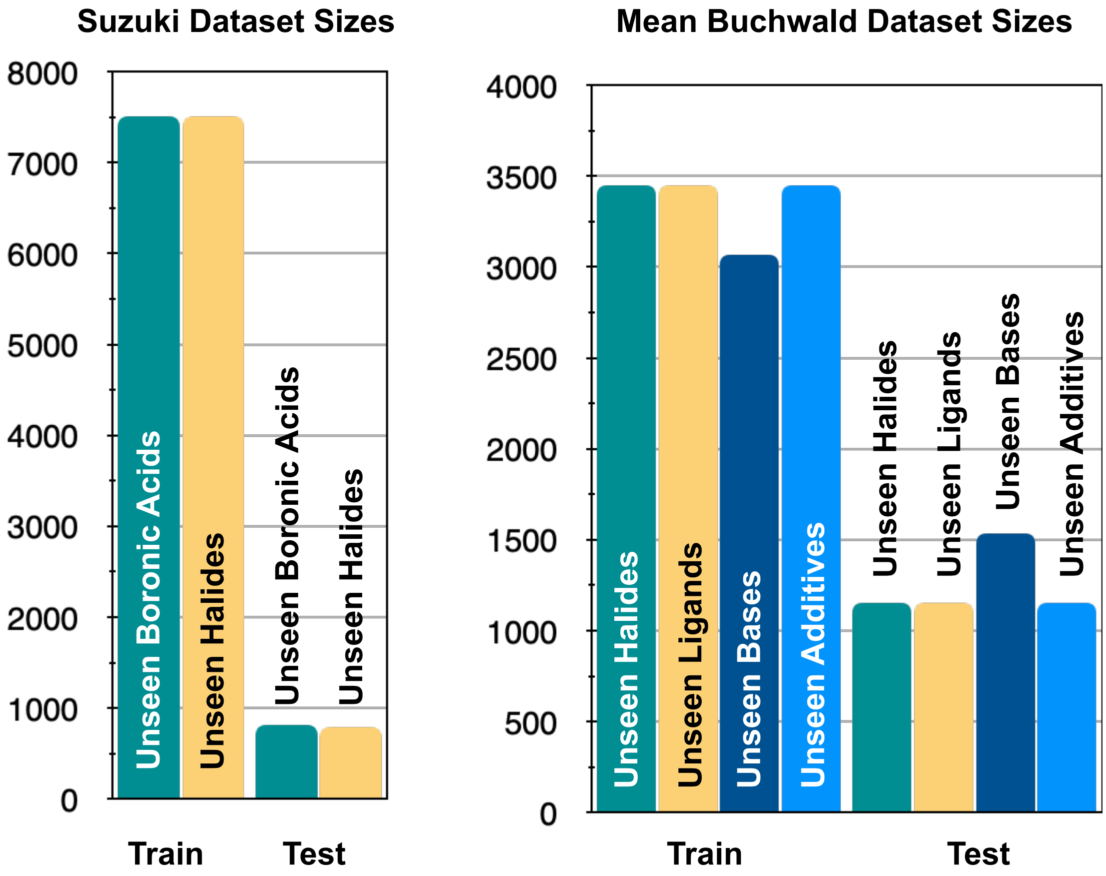

Figure S6: Training and testing dataset sizes for Suzuki and Buchwald-Hartwig coupling datasets. For the Buchwald-Hartwig couplings, the dataset size is the mean of each halide / ligand / base / additive being left out of the training set for testing.

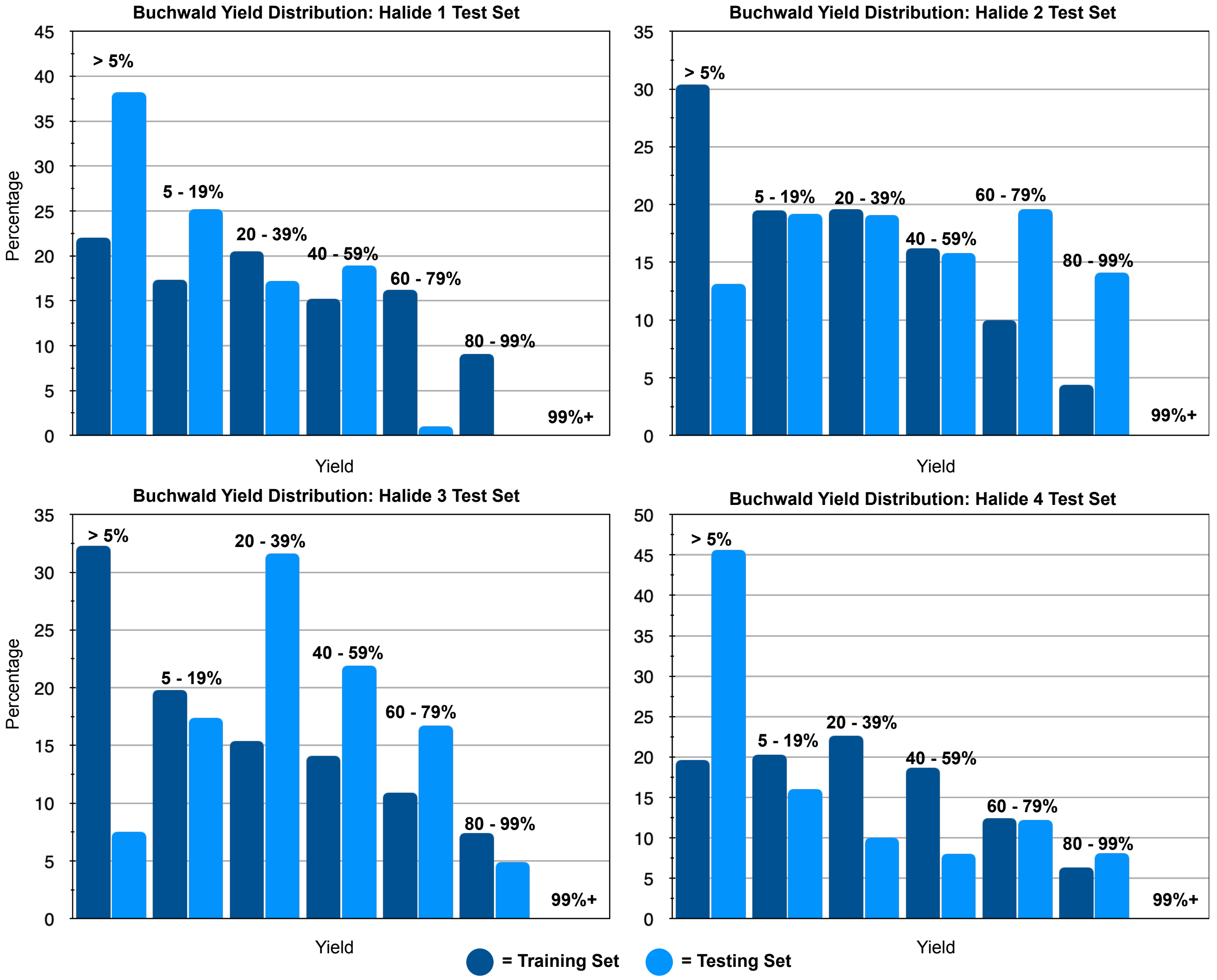

Figure S7: The product yield distributions for each halide test set. Note that these test sets combine 4 halides for a single split. Dark blue bars indicate training set distribution and light blue bars indicate testing set distribution.

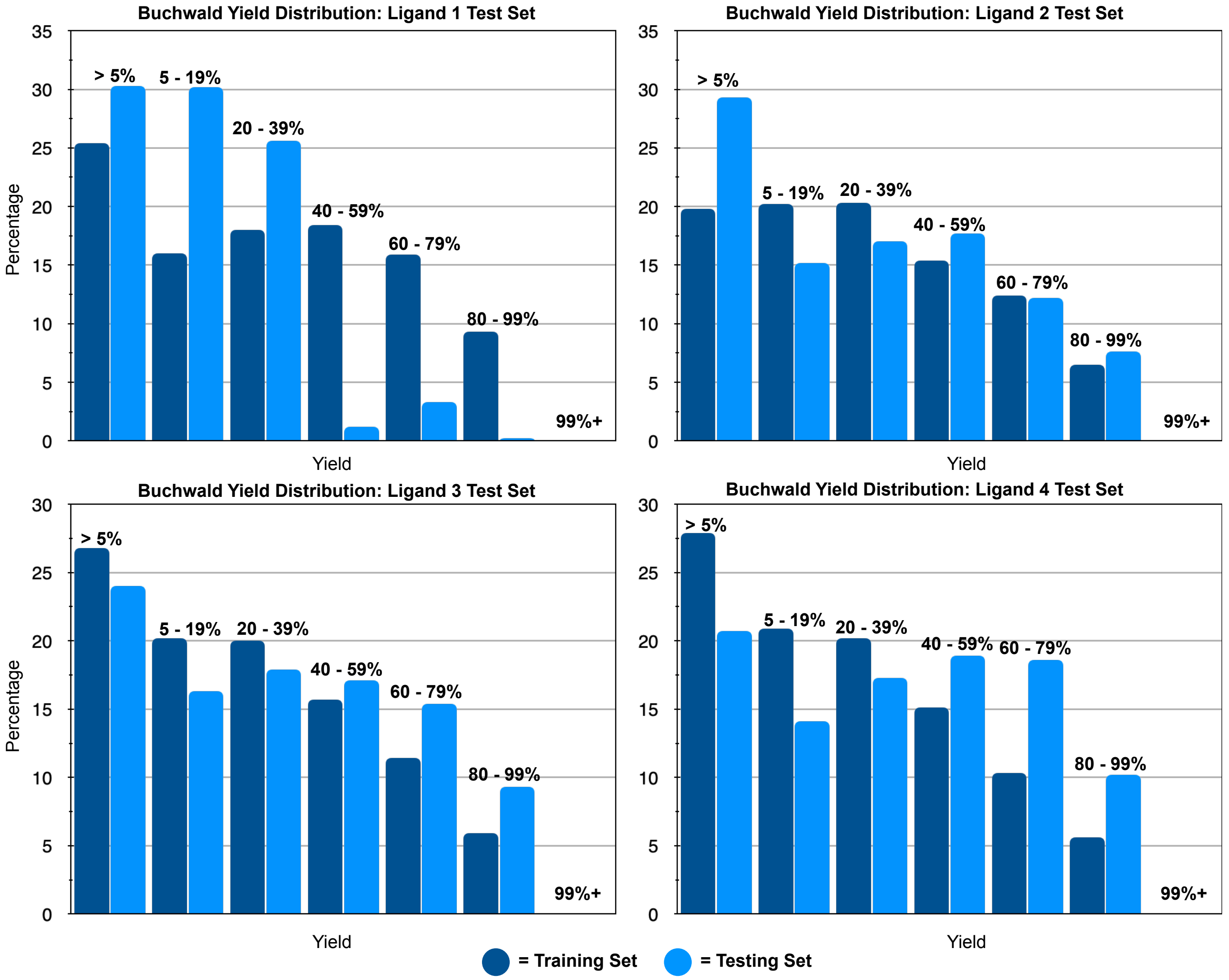

Figure S8: The product yield distributions for each ligand test set. Note that this test set uses one ligand per split. Dark blue bars indicate training set distribution and light blue bars indicate testing set distribution.

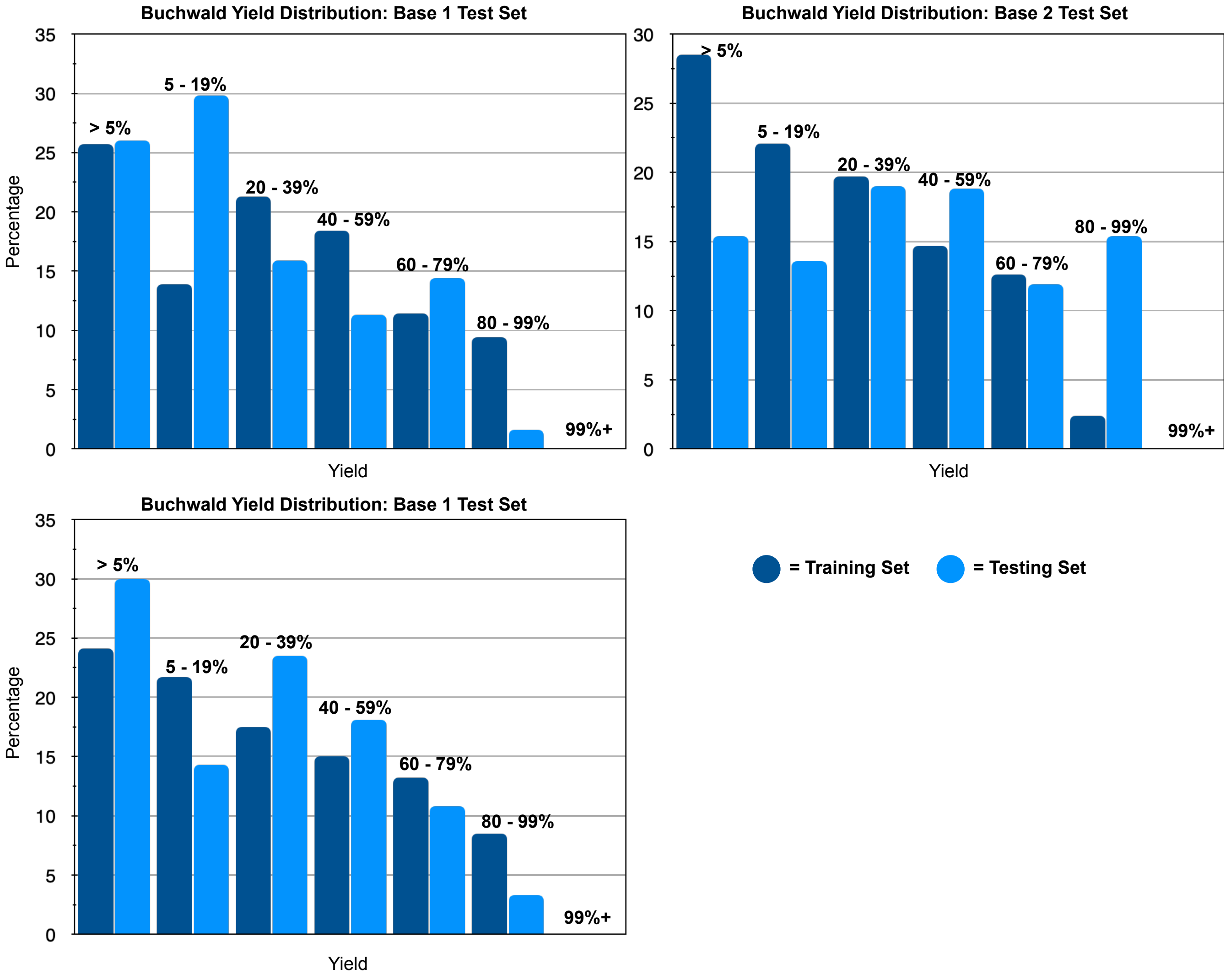

Figure S9: The product yield distributions for each base test set. Note that this test set uses one base per split. Dark blue bars indicate training set distribution and light blue bars indicate testing set distribution.

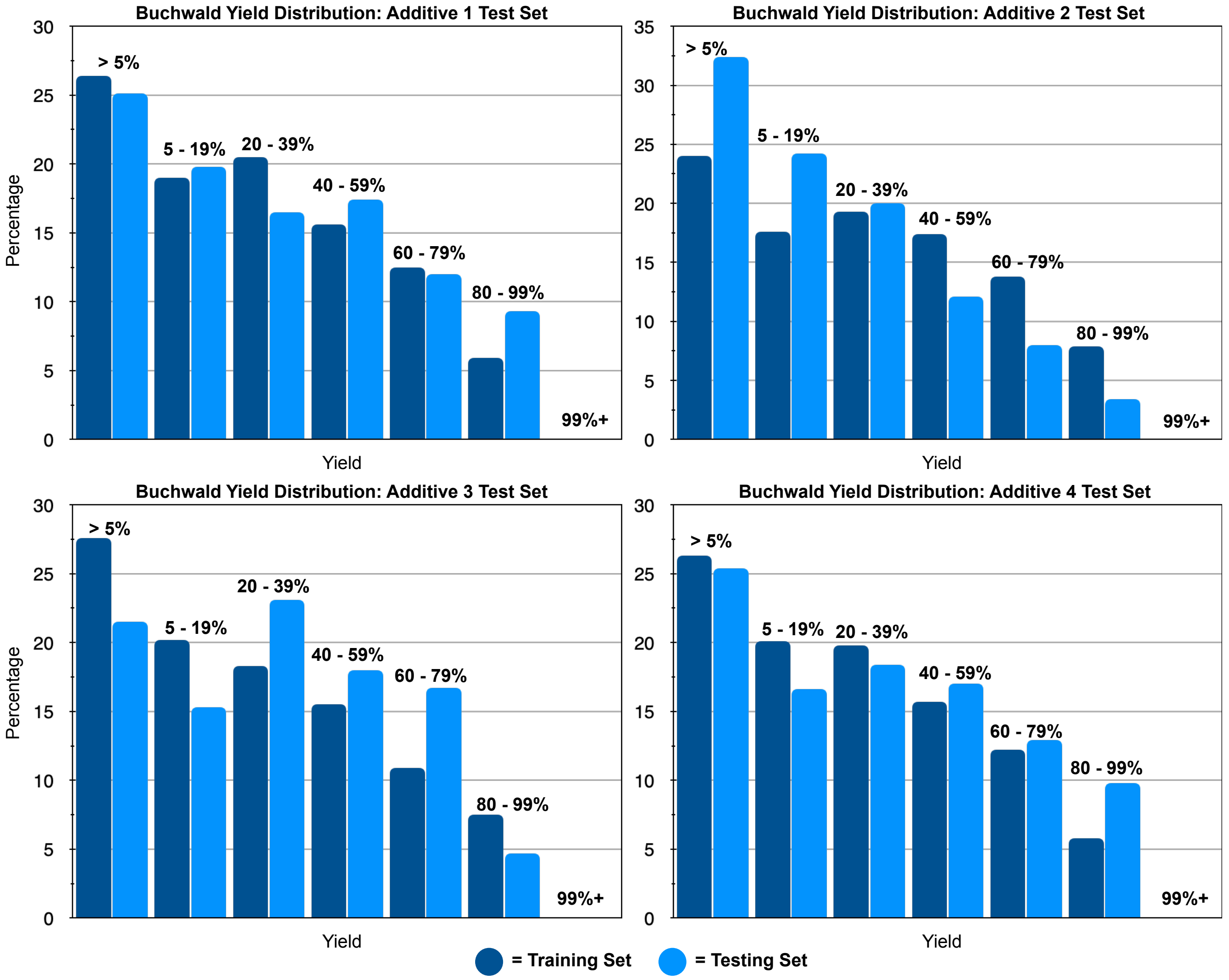

Figure S10: The product yield distributions for each additive test set. Note that these test sets combine 6 additives for a single split. Dark blue bars indicate training set distribution and light blue bars indicate testing set distribution.

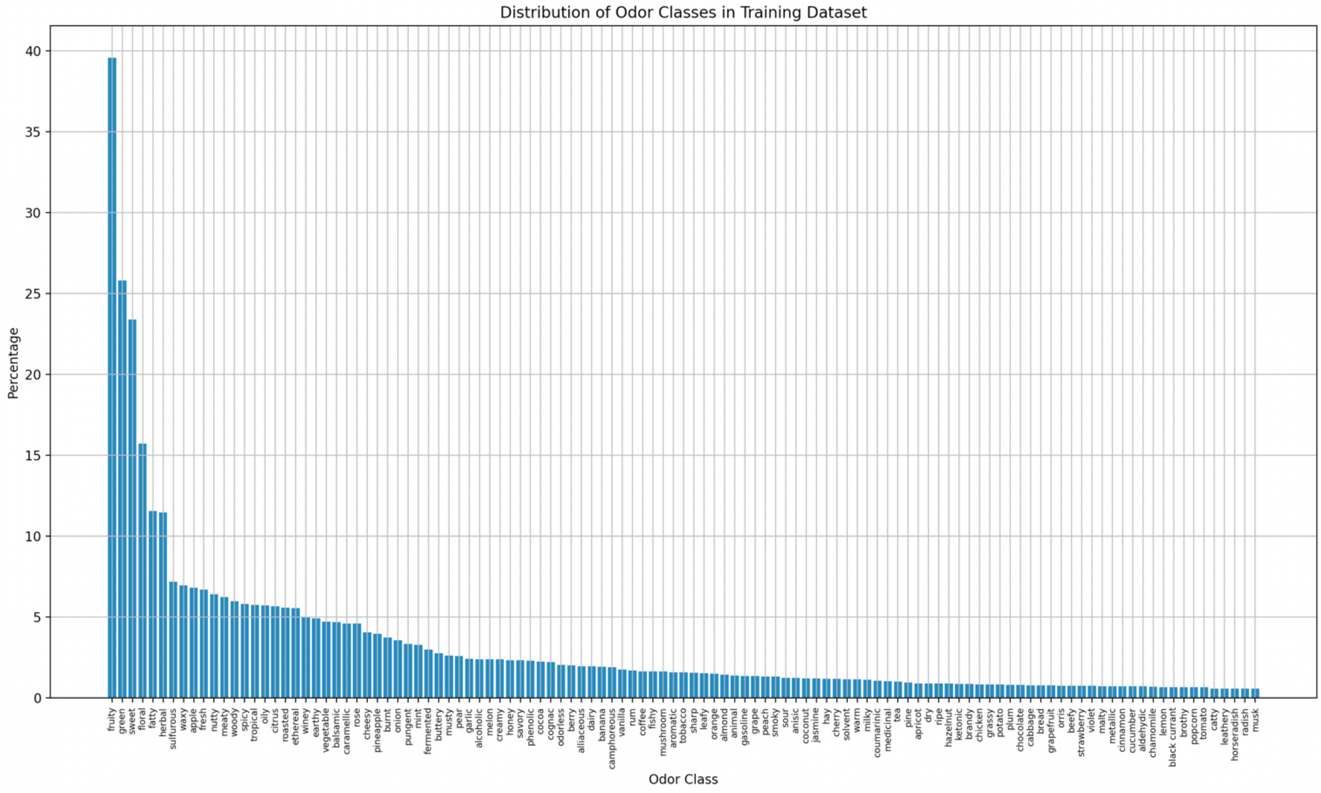

Figure S11: The complete breakdown of odor classes in the Pyrfume training dataset.

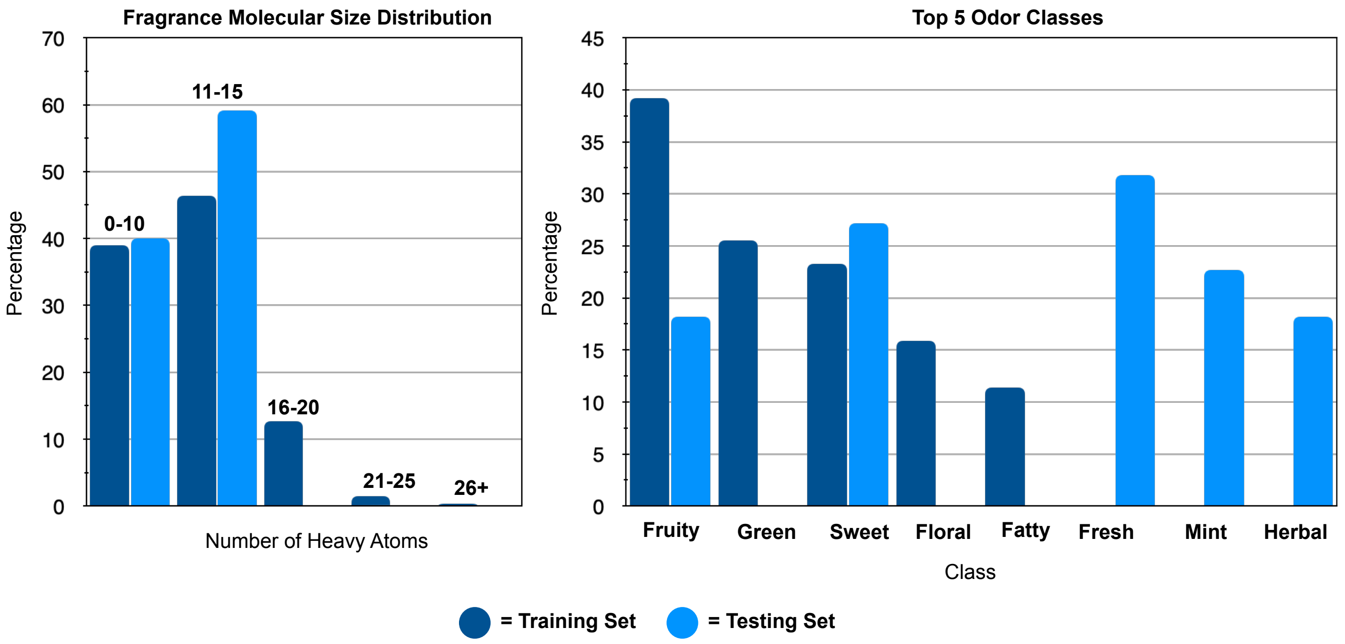

Figure S12: The difference between the fragrance training and enantiomeric pairs testing sets, in both molecular size and in the most common odor classification classes. Dark blue bars indicate training set distribution and light blue bars indicate testing set distribution.

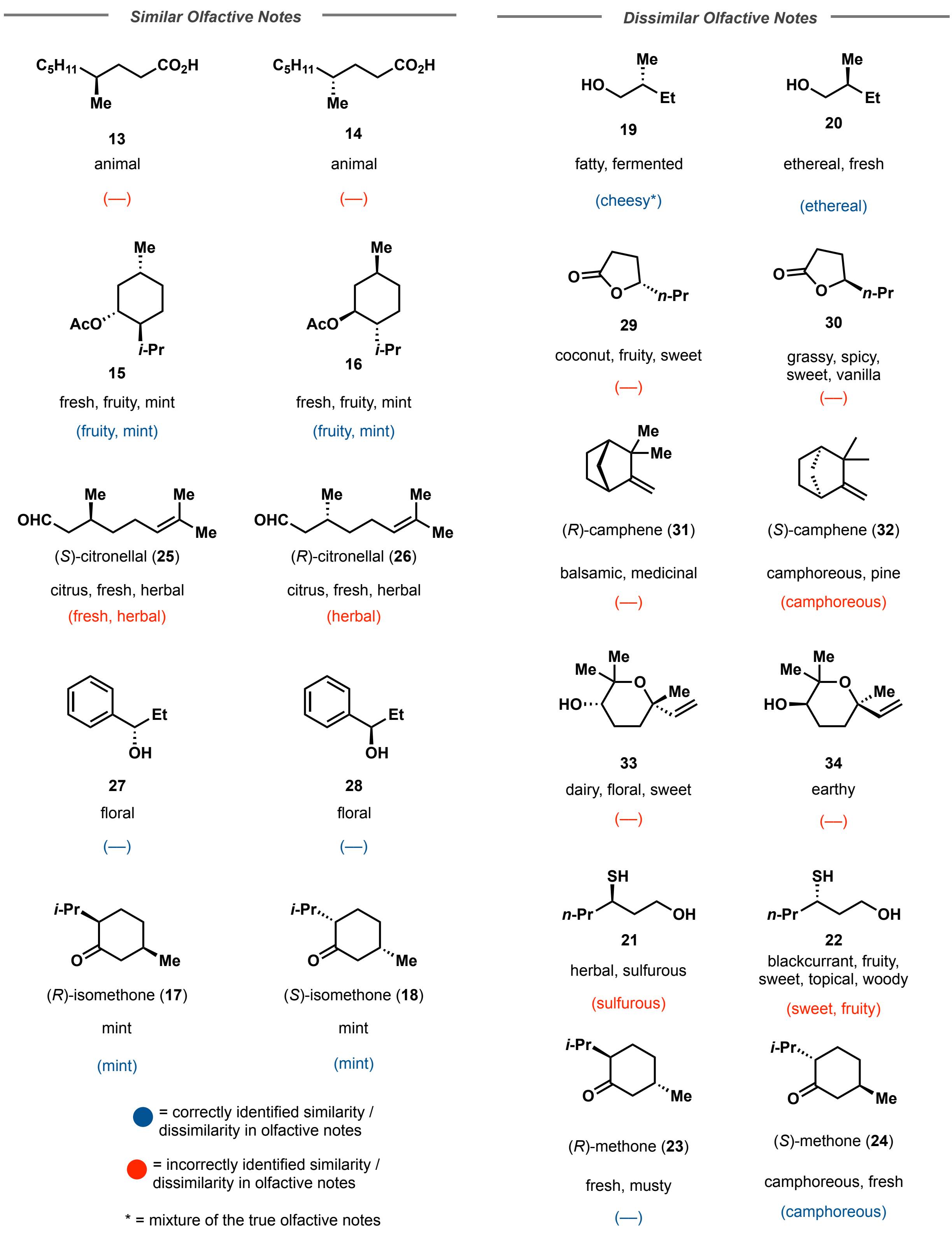

Figure S13: Predictions from best Crystal-Olfaction model on all enantiomeric pairs. For clarity, correct top 5 predictions are shown in parentheses. Horizontal line indicates that none of the top 5 most likely labels were correct. Blue predictions show that Crystal-Olfaction correctly identified that the enantiomeric pair had an identical / differing olfactive profile, even if no label was correctly predicted in the top 5. Red predictions indicate that Crystal-Olfaction determined incorrectly identified the similarity of scent profile between the enantiomeric pair.

User's Guide

This guide is written for non-experts looking to use the foundational model for transfer learning for their own tasks. We will begin with the basic installation and move into an example of how to use the foundational model for Buchwald-Hartwig yield prediction. The model was trained on Linux OS and we highly recommend that users do the same.

Prerequisites

Installation of Conda:
A good rule of thumb when working with code in Python is to use virtual environments for your projects. This will allow you to install the exact packages you need without worrying about conflicts from other projects / previous installations. We will be using conda virtual environments. Official instructions for installation can be found here: https://docs.conda.io/projects/miniconda/en/latest/. Please follow the instructions according to your operating system.

Installation of Git (Optional):
The program used to interface with GitHub is git. Although not a requirement for getting the model from the GitHub, it is a very useful tool in managing code. Official instructions for installation can be found here: https://github.com/git-guides/install-git. Please follow the instructions according to your operating system.

Getting the Code

We will show you the commands used (highlighted in yellow) and the associated output (the line after the command is run) to assist users in using command line inputs and our model. Commands can be run by pressing the "return" key. Within this text, commands will be indicated with `this font`. These commands are to be implemented in your computer's terminal / console.

Create a New Directory:
A directory can also be referred to as a "folder". For the remainder of the document, we will be using the term directory but you may think of them as folders. To access our code, two methods are possible. The first uses git, the second goes through GitHub.com's web-interface. We will go through both.

Start by making a new directory called "transfer_learning" and move into the directory. This can be done by typing:

```
mkdir transfer_learning
```

There will be no output from this command.

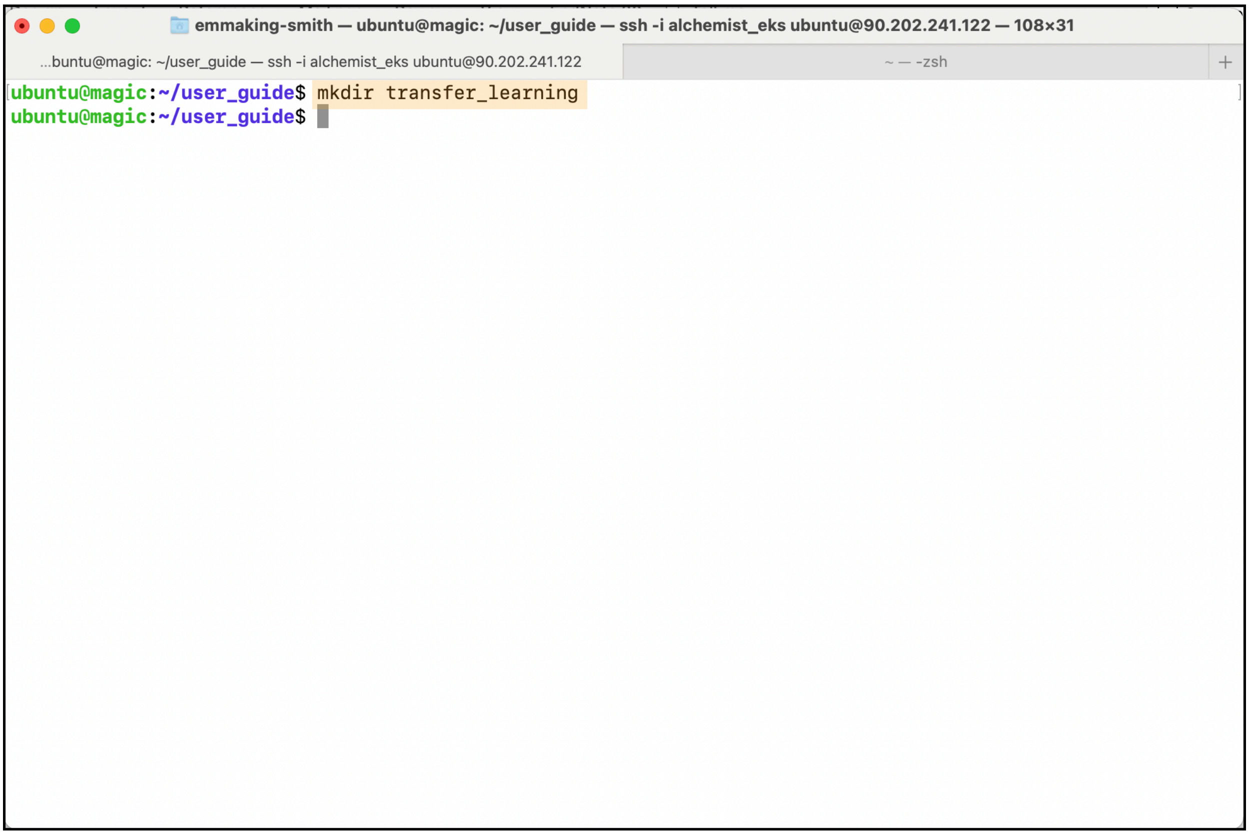

Move into the directory with:

```
cd transfer_learning
```

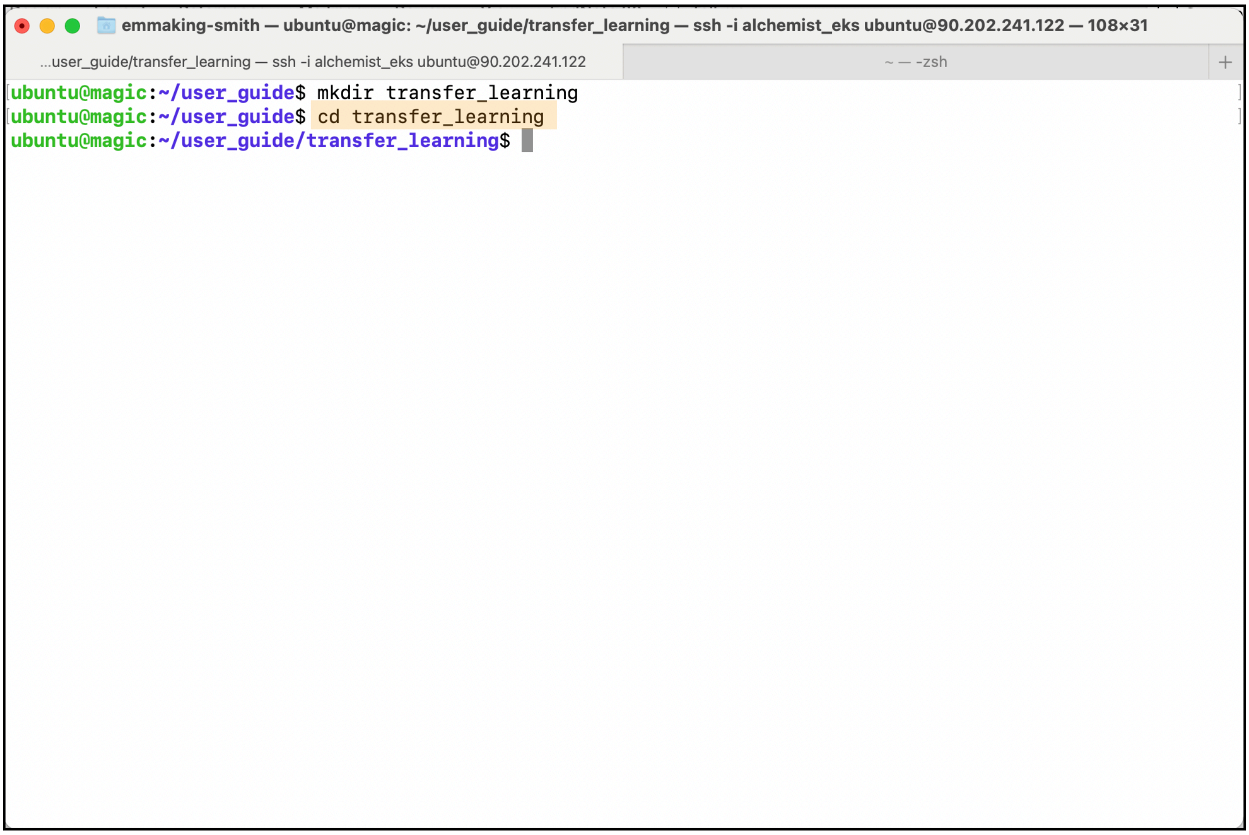

We are now ready to download the code for Transfer Learning for a Foundational Chemistry Model. You have two options. Option 1 requires the installation of git and Option 2 does not. Please choose **either** Option 1 or Option 2.

Option 1 - Download the GitHub Repository with Git (Git installation required):

Clone the repository. This allows you to instantly download all the information off of the repository to your new directory. Type:

```
git clone https://github.com/emmaking-smith/Modular_Latent_Space.git
```

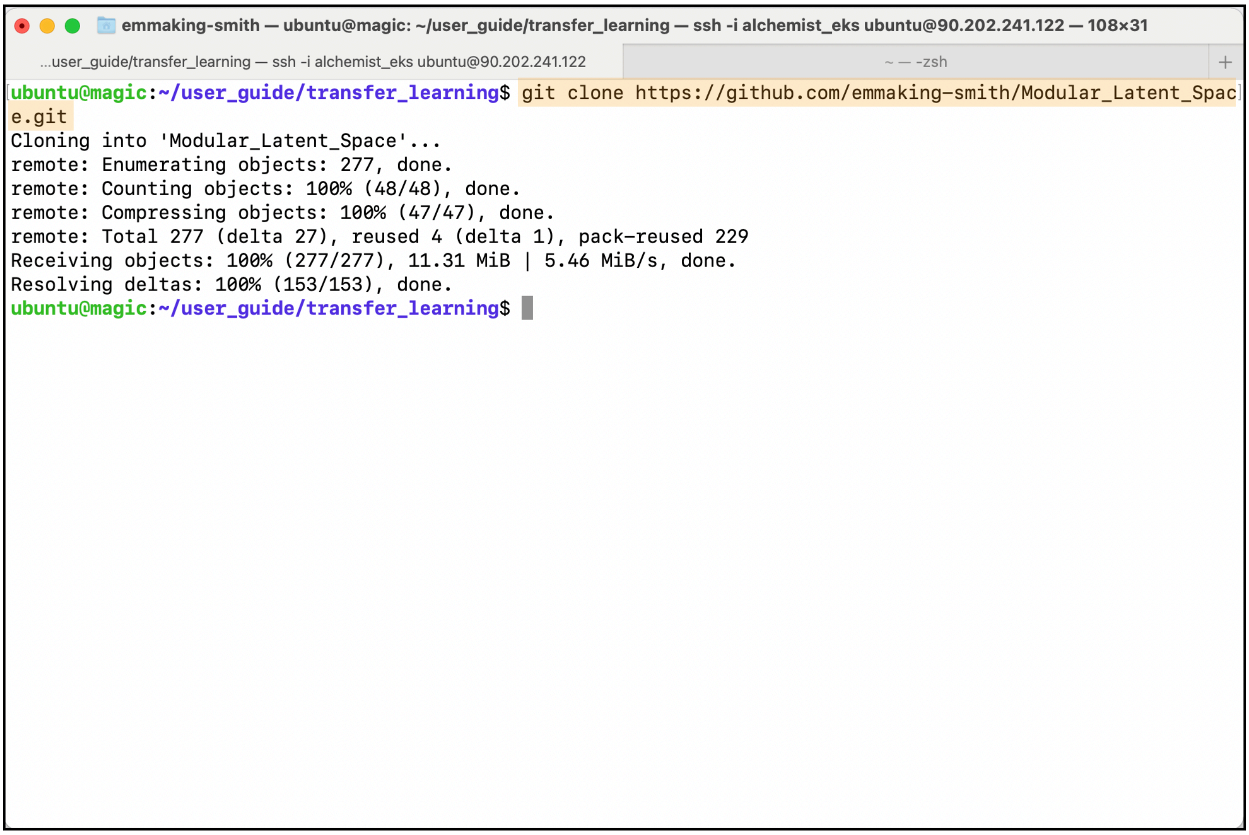

Option 2 - Download the GitHub Repository from GitHub.com:

If you do not want to install git, you may download all the code from the GitHub website.
Go to https://github.com/emmaking-smith/Modular_Latent_Space.

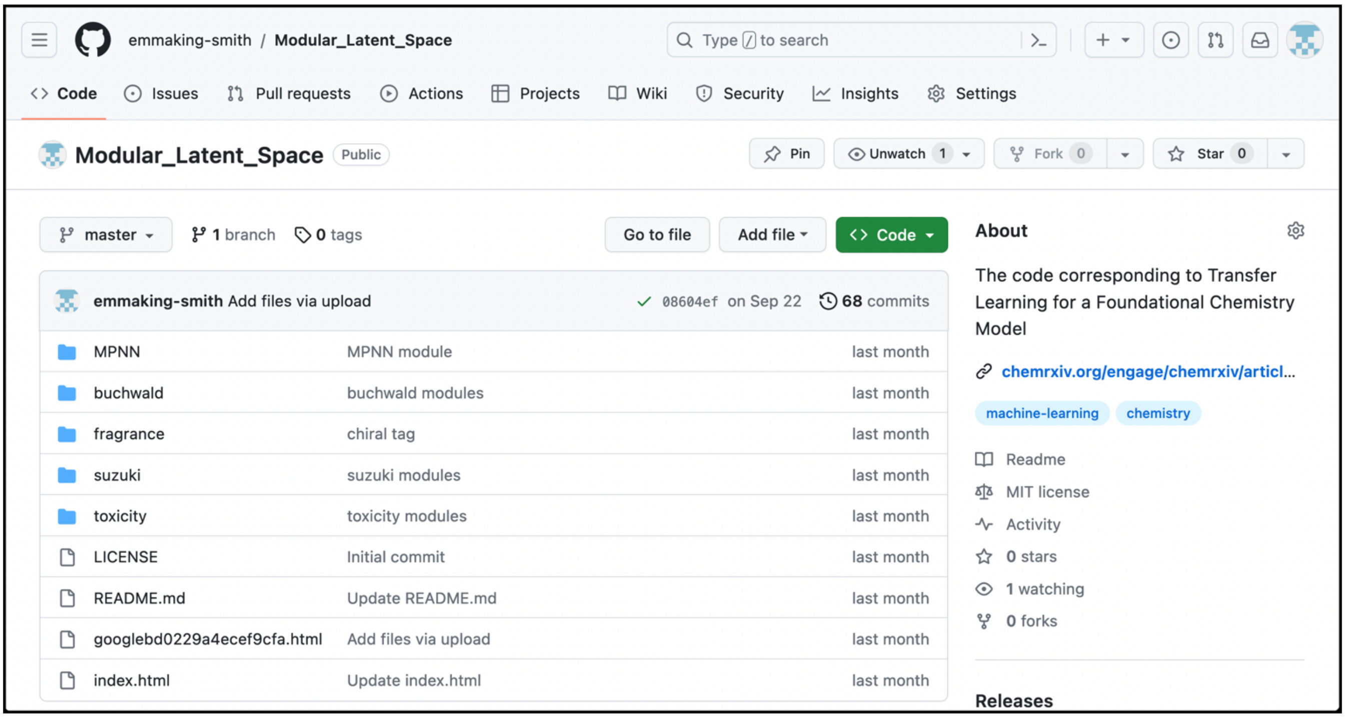

Click on the green "Code" button.

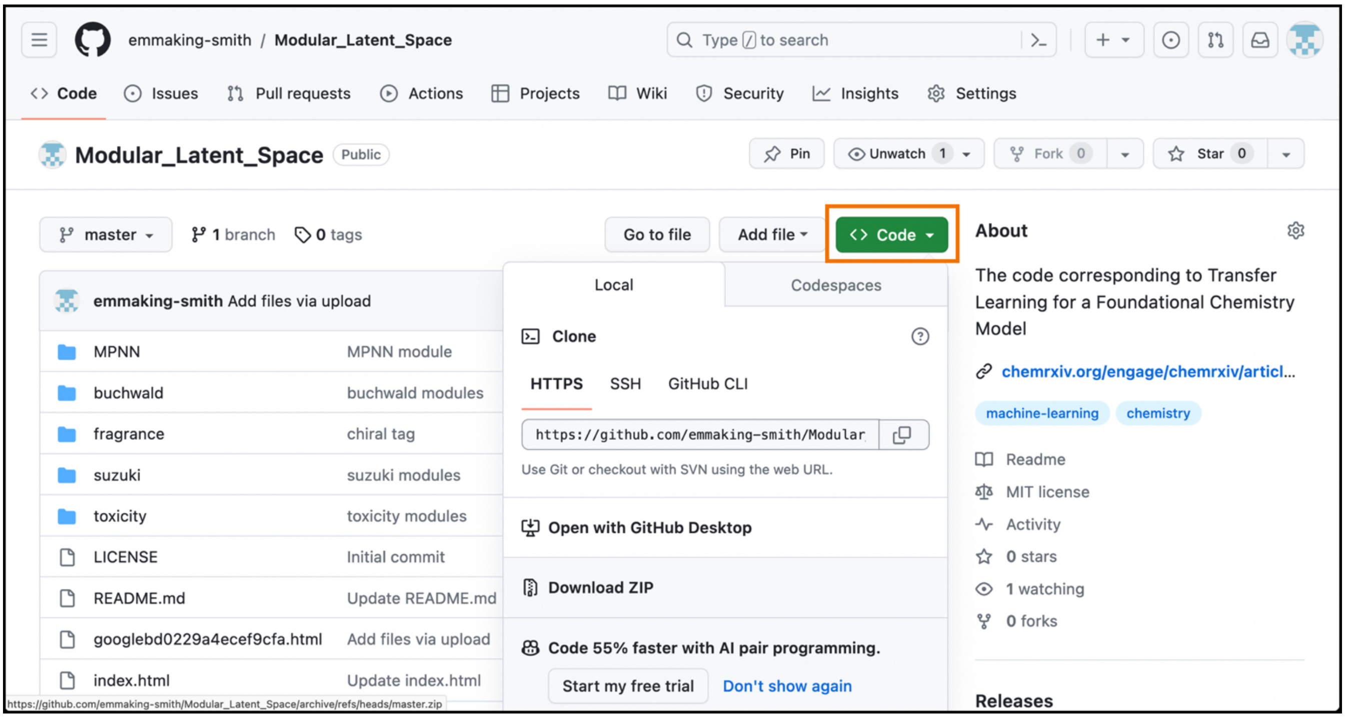

Select "Download ZIP"

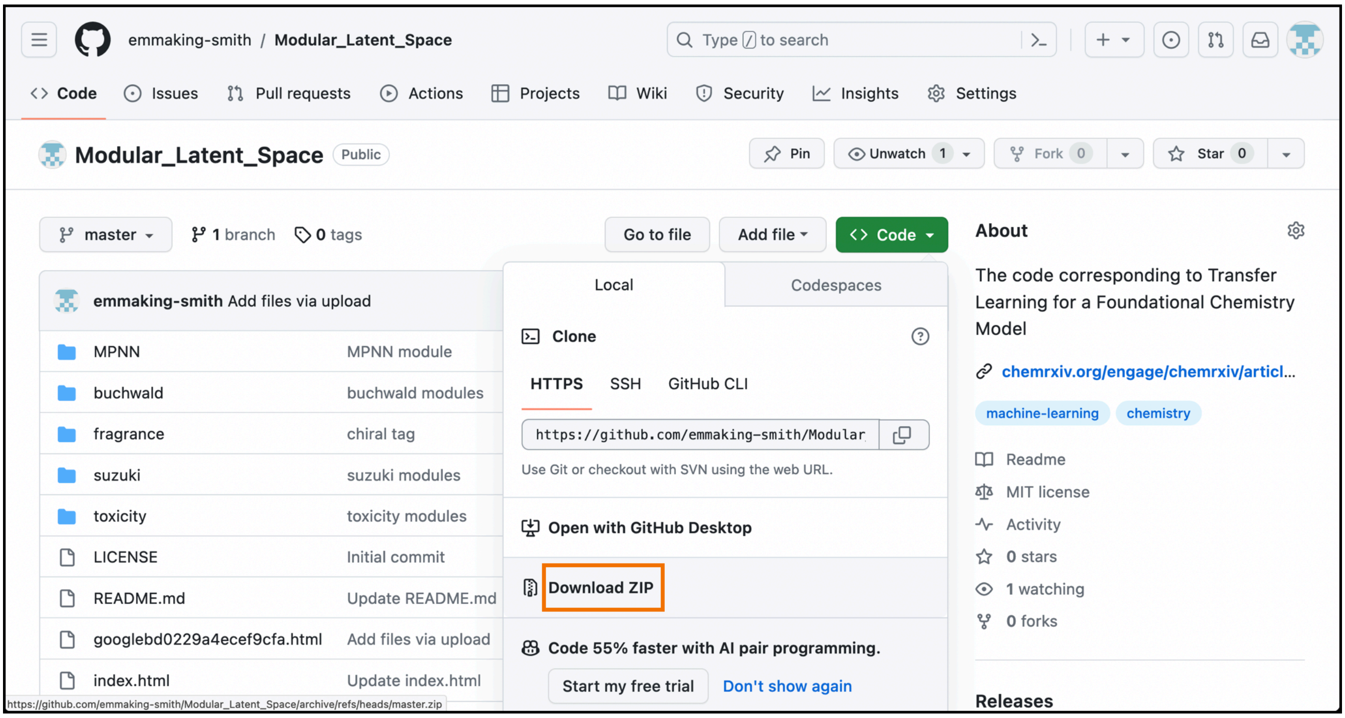

Unzip the zip file. This can typically be achieved by double clicking on the file.

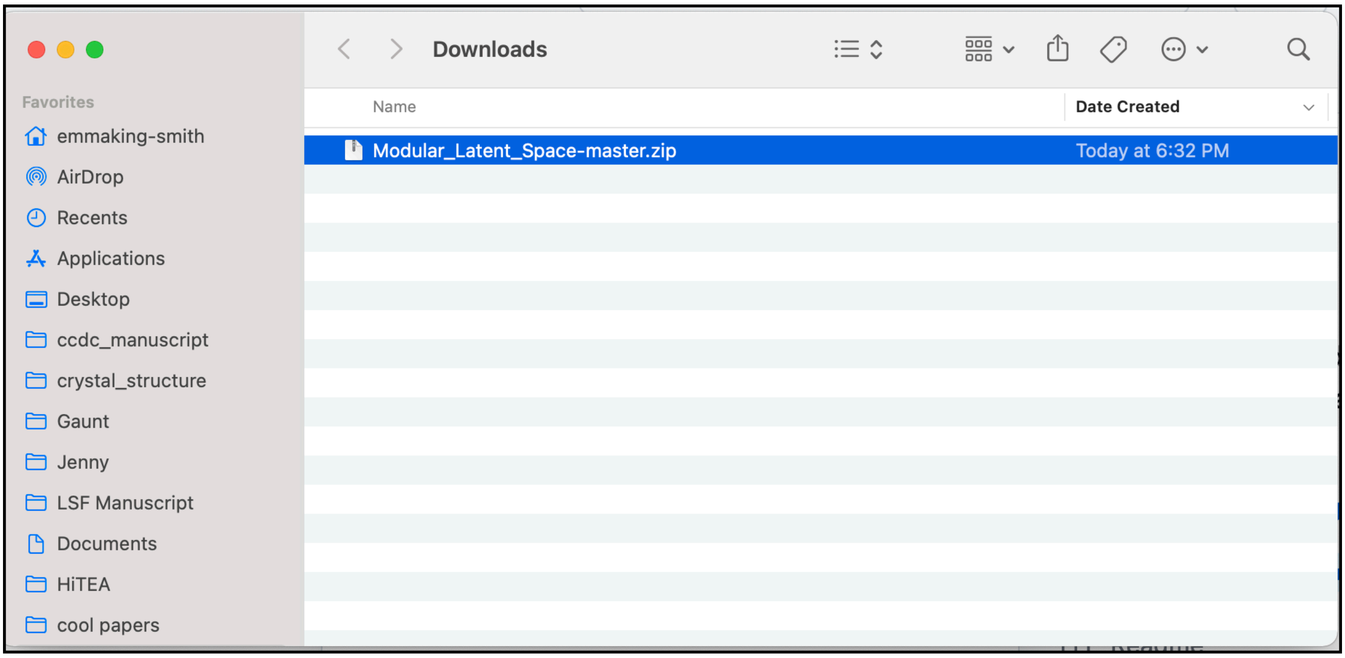

Move the unzipped directory, Modular_Latent_Space-master from its current directory to the directory we created at the beginning of this section (we named it transfer_learning). Drag and drop is the easiest way to do this.

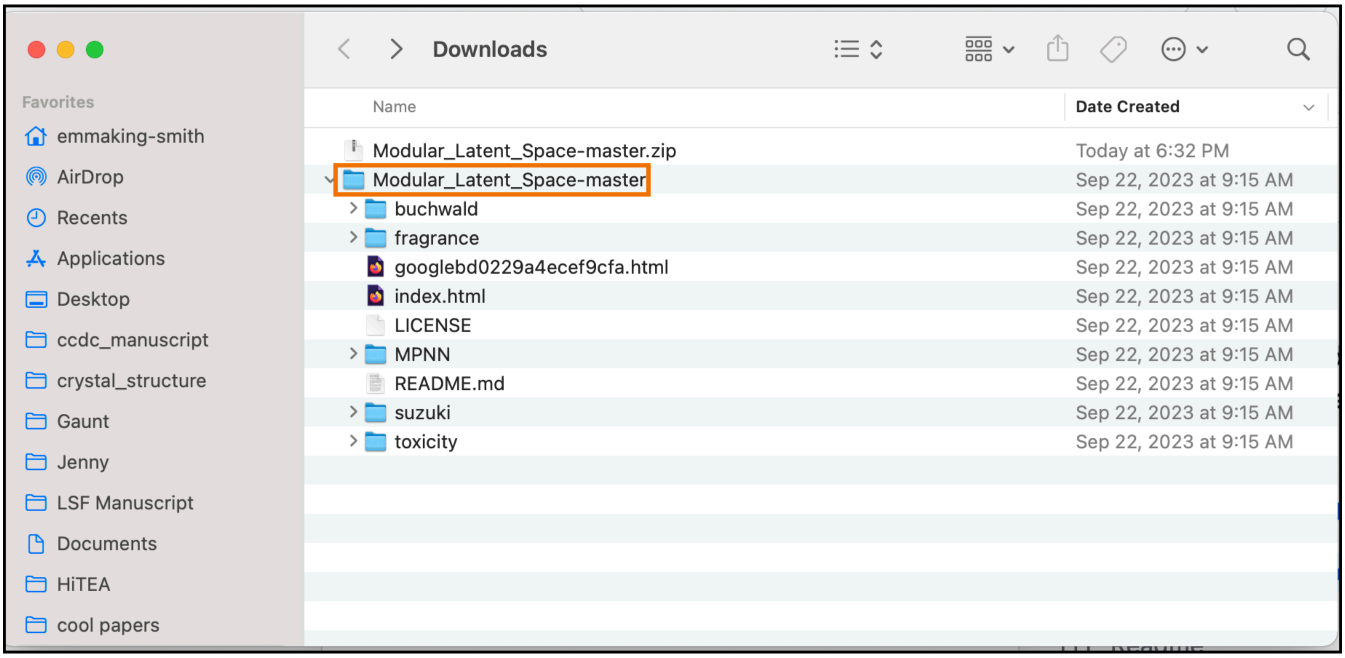

Setting up the Virtual Environment

Here, we install the necessary packages to run the transfer learning. The necessary packages can be found at https://github.com/emmaking-smith/Modular_Latent_Space at the bottom of the page under "Dependencies".

First we create our virtual environment that will be used to run all of our code. Note that you must be within this virtual environment to run the code without errors. To do this, we run the following command:

```
conda create -n tl3.7 python==3.7
```

Our virtual environment's name is tl3.7 (transfer learning python version 3.7). We designate the python version with the suffix of "`python==3.7`". **It is critical to use the correct version of python when attempting to use any other programmer's code.**

```
emmaking-smith — ubuntu@magic: ~/user_guide — ssh -i alchemist_eks ubuntu@90.202.241.122 — 108×31
...buntu@magic: ~/user_guide — ssh -i alchemist_eks ubuntu@90.202.241.122          ~ — -zsh
ubuntu@magic:~/user_guide$ conda create -n tl3.7 python==3.7
Collecting package metadata (current_repodata.json): done
Solving environment: unsuccessful attempt using repodata from current_repodata.json, retrying with next repo
data source.
Collecting package metadata (repodata.json): done
Solving environment: done


==> WARNING: A newer version of conda exists. <==
  current version: 23.7.3
  latest version: 23.9.0

Please update conda by running

    $ conda update -n base -c defaults conda

Or to minimize the number of packages updated during conda update use

     conda install conda=23.9.0



## Package Plan ##

  environment location: /home/ubuntu/miniconda3/envs/tl3.7

  added / updated specs:
    - python==3.7


The following NEW packages will be INSTALLED:
```

Shortly thereafter, the console will prompt you to accept the installation of new packages. Type:

y

```
emmaking-smith — ubuntu@magic: ~/user_guide — ssh -i alchemist_eks ubuntu@90.202.241.122 — 108×31
...buntu@magic: ~/user_guide — ssh -i alchemist_eks ubuntu@90.202.241.122          ~ — -zsh
  environment location: /home/ubuntu/miniconda3/envs/tl3.7

  added / updated specs:
    - python==3.7


The following NEW packages will be INSTALLED:

  _libgcc_mutex      pkgs/main/linux-64::_libgcc_mutex-0.1-main
  _openmp_mutex      pkgs/main/linux-64::_openmp_mutex-5.1-1_gnu
  ca-certificates    pkgs/main/linux-64::ca-certificates-2023.08.22-h06a4308_0
  certifi            pkgs/main/linux-64::certifi-2022.12.7-py37h06a4308_0
  libedit            pkgs/main/linux-64::libedit-3.1.20221030-h5eee18b_0
  libffi             pkgs/main/linux-64::libffi-3.2.1-hf484d3e_1007
  libgcc-ng          pkgs/main/linux-64::libgcc-ng-11.2.0-h1234567_1
  libgomp            pkgs/main/linux-64::libgomp-11.2.0-h1234567_1
  libstdcxx-ng       pkgs/main/linux-64::libstdcxx-ng-11.2.0-h1234567_1
  ncurses            pkgs/main/linux-64::ncurses-6.4-h6a678d5_0
  openssl            pkgs/main/linux-64::openssl-1.0.2u-h7b6447c_0
  pip                pkgs/main/linux-64::pip-22.3.1-py37h06a4308_0
  python             pkgs/main/linux-64::python-3.7.0-h6e4f718_3
  readline           pkgs/main/linux-64::readline-7.0-h7b6447c_5
  setuptools         pkgs/main/linux-64::setuptools-65.6.3-py37h06a4308_0
  sqlite             pkgs/main/linux-64::sqlite-3.33.0-h62c20be_0
  tk                 pkgs/main/linux-64::tk-8.6.12-h1ccaba5_0
  wheel              pkgs/main/linux-64::wheel-0.38.4-py37h06a4308_0
  xz                 pkgs/main/linux-64::xz-5.4.2-h5eee18b_0
  zlib               pkgs/main/linux-64::zlib-1.2.13-h5eee18b_0


Proceed ([y]/n)? y
```

The output of these commands will look something like this:

```
emmaking-smith — ubuntu@magic: ~/user_guide — ssh -i alchemist_eks ubuntu@90.202.241.122 — 108×31
...buntu@magic: ~/user_guide — ssh -i alchemist_eks ubuntu@90.202.241.122        ~ — -zsh
  ncurses            pkgs/main/linux-64::ncurses-6.4-h6a678d5_0
  openssl            pkgs/main/linux-64::openssl-1.0.2u-h7b6447c_0
  pip                pkgs/main/linux-64::pip-22.3.1-py37h06a4308_0
  python             pkgs/main/linux-64::python-3.7.0-h6e4f718_3
  readline           pkgs/main/linux-64::readline-7.0-h7b6447c_5
  setuptools         pkgs/main/linux-64::setuptools-65.6.3-py37h06a4308_0
  sqlite             pkgs/main/linux-64::sqlite-3.33.0-h62c20be_0
  tk                 pkgs/main/linux-64::tk-8.6.12-h1ccaba5_0
  wheel              pkgs/main/linux-64::wheel-0.38.4-py37h06a4308_0
  xz                 pkgs/main/linux-64::xz-5.4.2-h5eee18b_0
  zlib               pkgs/main/linux-64::zlib-1.2.13-h5eee18b_0


Proceed ([y]/n)? y


Downloading and Extracting Packages

Preparing transaction: done
Verifying transaction: done
Executing transaction: done
#
# To activate this environment, use
#
#     $ conda activate tl3.7
#
# To deactivate an active environment, use
#
#     $ conda deactivate

ubuntu@magic:~/user_guide$
```

We then activate our virtual environment.

```
conda activate tl3.7
```

You can tell that you are in a virtual environment by the leftmost text, which now says the environment name (see orange box).

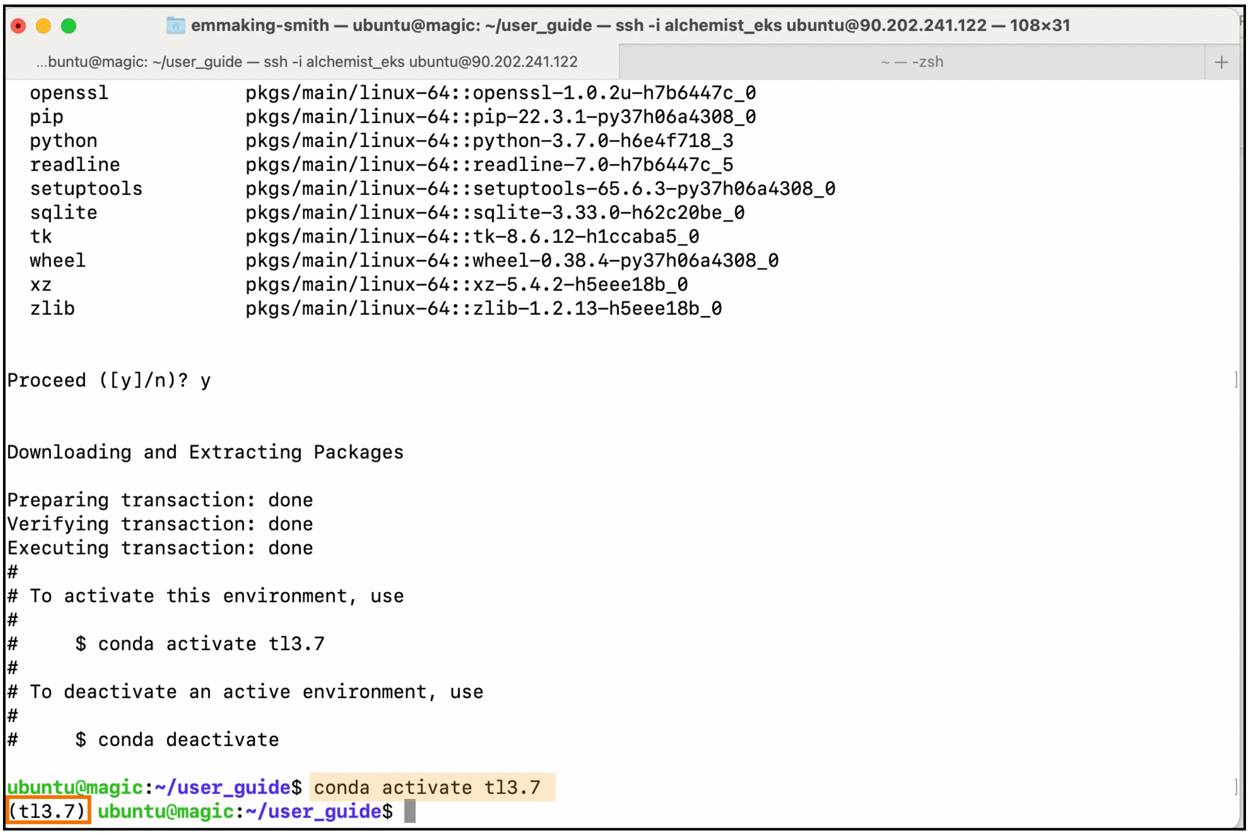

emmaking-smith — ubuntu@magic: ~/user_guide — ssh -i alchemist_eks ubuntu@90.202.241.122 — 108×31

...buntu@magic: ~/user_guide — ssh -i alchemist_eks ubuntu@90.202.241.122 | ~ — -zsh

```
  openssl            pkgs/main/linux-64::openssl-1.0.2u-h7b6447c_0
  pip                pkgs/main/linux-64::pip-22.3.1-py37h06a4308_0
  python             pkgs/main/linux-64::python-3.7.0-h6e4f718_3
  readline           pkgs/main/linux-64::readline-7.0-h7b6447c_5
  setuptools         pkgs/main/linux-64::setuptools-65.6.3-py37h06a4308_0
  sqlite             pkgs/main/linux-64::sqlite-3.33.0-h62c20be_0
  tk                 pkgs/main/linux-64::tk-8.6.12-h1ccaba5_0
  wheel              pkgs/main/linux-64::wheel-0.38.4-py37h06a4308_0
  xz                 pkgs/main/linux-64::xz-5.4.2-h5eee18b_0
  zlib               pkgs/main/linux-64::zlib-1.2.13-h5eee18b_0


Proceed ([y]/n)? y


Downloading and Extracting Packages

Preparing transaction: done
Verifying transaction: done
Executing transaction: done
#
# To activate this environment, use
#
#     $ conda activate tl3.7
#
# To deactivate an active environment, use
#
#     $ conda deactivate

ubuntu@magic:~/user_guide$ conda activate tl3.7
(tl3.7) ubuntu@magic:~/user_guide$
```

Package Installation

Next, all the relevant packages of specific versions will be installed. We specify this with the "==" sign. RDKit will be installed first.

```
conda install -y -c rdkit rdkit==2020.09.1
```

```
emmaking-smith — ubuntu@magic: ~/user_guide/transfer_learning/Modular_Latent_Space — ssh -i alchemist_eks ubuntu@90.202.241.12...
~ — -zsh    .../Modular_Latent_Space — ssh -i alchemist_eks ubuntu@90.202.241.122
ubuntu@magic:~/user_guide/transfer_learning/Modular_Latent_Space$ conda activate tl3.7
(tl3.7) ubuntu@magic:~/user_guide/transfer_learning/Modular_Latent_Space$ conda install -y -c rdkit rdkit==2
020.09.1
Collecting package metadata (current_repodata.json): done
Solving environment: unsuccessful initial attempt using frozen solve. Retrying with flexible solve.
Solving environment: unsuccessful attempt using repodata from current_repodata.json, retrying with next repo
data source.
Collecting package metadata (repodata.json): done
Solving environment: done


==> WARNING: A newer version of conda exists. <==
  current version: 23.7.3
  latest version: 23.9.0

Please update conda by running

    $ conda update -n base -c defaults conda

Or to minimize the number of packages updated during conda update use

     conda install conda=23.9.0



## Package Plan ##

  environment location: /home/ubuntu/miniconda3/envs/tl3.7

  added / updated specs:
    - rdkit==2020.09.1
```

You will see many packages being installed. The final output will look something like this:

```
emmaking-smith — ubuntu@magic: ~/user_guide/transfer_learning/Modular_Latent_Space — ssh -i alchemist_eks ubuntu@90.202.241.12...
~ — -zsh    .../Modular_Latent_Space — ssh -i alchemist_eks ubuntu@90.202.241.122
  libwebp-base       pkgs/main/linux-64::libwebp-base-1.2.4-h5eee18b_1
  libxcb             pkgs/main/linux-64::libxcb-1.15-h7f8727e_0
  libxml2            pkgs/main/linux-64::libxml2-2.10.4-hcbfbd50_0
  lz4-c              pkgs/main/linux-64::lz4-c-1.9.4-h6a678d5_0
  mkl                pkgs/main/linux-64::mkl-2021.4.0-h06a4308_640
  mkl-service        pkgs/main/linux-64::mkl-service-2.4.0-py37h7f8727e_0
  mkl_fft            pkgs/main/linux-64::mkl_fft-1.3.1-py37hd3c417c_0
  mkl_random         pkgs/main/linux-64::mkl_random-1.2.2-py37h51133e4_0
  numexpr            pkgs/main/linux-64::numexpr-2.8.4-py37he184ba9_0
  numpy              pkgs/main/linux-64::numpy-1.21.5-py37h6c91a56_3
  numpy-base         pkgs/main/linux-64::numpy-base-1.21.5-py37ha15fc14_3
  packaging          pkgs/main/linux-64::packaging-22.0-py37h06a4308_0
  pandas             pkgs/main/linux-64::pandas-1.3.5-py37h8c16a72_0
  pcre               pkgs/main/linux-64::pcre-8.45-h295c915_0
  pillow             pkgs/main/linux-64::pillow-9.4.0-py37h6a678d5_0
  pixman             pkgs/main/linux-64::pixman-0.40.0-h7f8727e_1
  py-boost           pkgs/main/linux-64::py-boost-1.73.0-py37h51133e4_12
  python-dateutil    pkgs/main/noarch::python-dateutil-2.8.2-pyhd3eb1b0_0
  pytz               pkgs/main/linux-64::pytz-2022.7-py37h06a4308_0
  rdkit              rdkit/linux-64::rdkit-2020.09.1.0-py37hd50e099_1
  six                pkgs/main/noarch::six-1.16.0-pyhd3eb1b0_1
  zstd               pkgs/main/linux-64::zstd-1.5.5-hc292b87_0



Downloading and Extracting Packages

Preparing transaction: done
Verifying transaction: done
Executing transaction: done
(tl3.7) ubuntu@magic:~/user_guide/transfer_learning/Modular_Latent_Space$
```

Pytorch and other useful torch packages are installed next with:

```
pip3 install torch==1.10.0+cu113 torchvision==0.11.1+cu113
torchaudio===0.10.0+cu113 -f
https://download.pytorch.org/whl/cu113/torch_stable.html
```

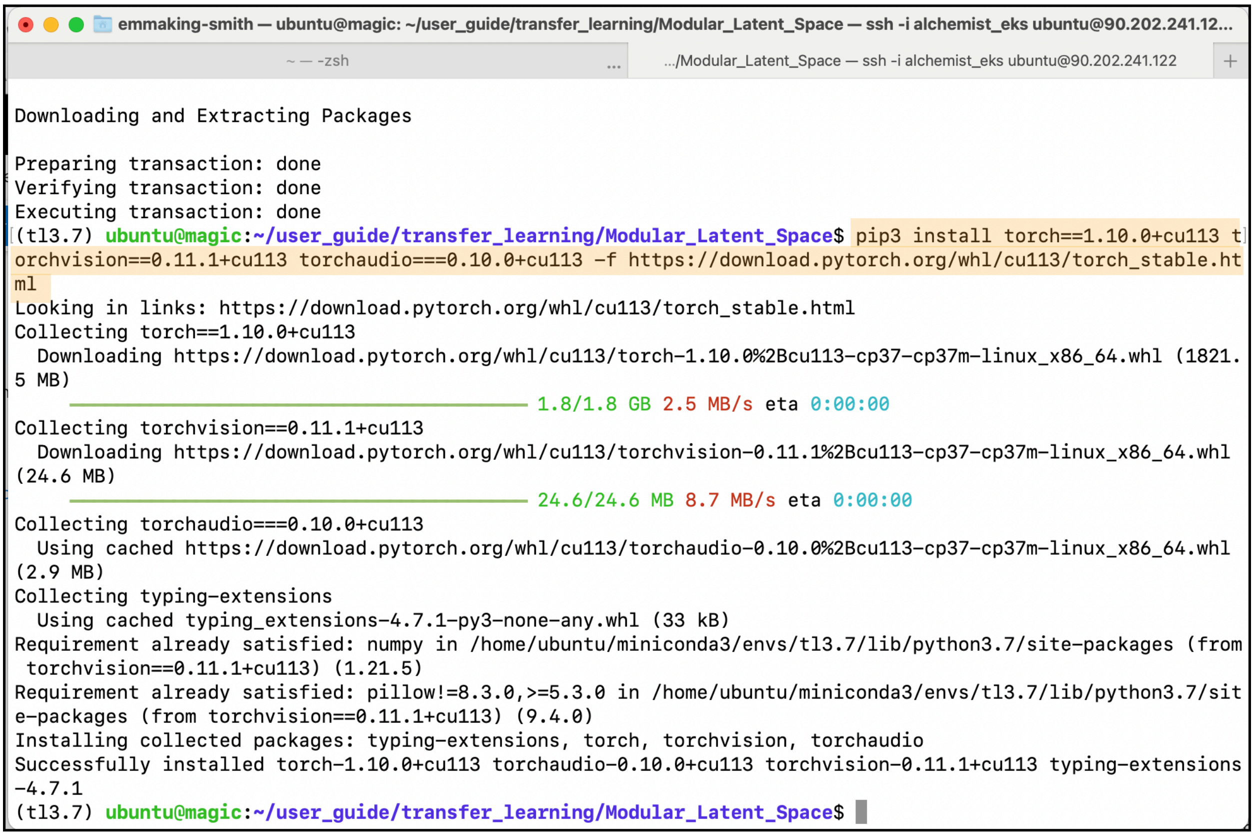

```
emmaking-smith — ubuntu@magic: ~/user_guide/transfer_learning/Modular_Latent_Space — ssh -i alchemist_eks ubuntu@90.202.241.12...
~ — -zsh    .../Modular_Latent_Space — ssh -i alchemist_eks ubuntu@90.202.241.122

Downloading and Extracting Packages

Preparing transaction: done
Verifying transaction: done
Executing transaction: done
(tl3.7) ubuntu@magic:~/user_guide/transfer_learning/Modular_Latent_Space$ pip3 install torch==1.10.0+cu113 t
orchvision==0.11.1+cu113 torchaudio===0.10.0+cu113 -f https://download.pytorch.org/whl/cu113/torch_stable.ht
ml
Looking in links: https://download.pytorch.org/whl/cu113/torch_stable.html
Collecting torch==1.10.0+cu113
  Downloading https://download.pytorch.org/whl/cu113/torch-1.10.0%2Bcu113-cp37-cp37m-linux_x86_64.whl (1821.
5 MB)
     ━━━━━━━━━━━━━━━━━━━━━━━━━━━━━━━━━━━━━━━━ 1.8/1.8 GB 2.5 MB/s eta 0:00:00
Collecting torchvision==0.11.1+cu113
  Downloading https://download.pytorch.org/whl/cu113/torchvision-0.11.1%2Bcu113-cp37-cp37m-linux_x86_64.whl
(24.6 MB)
     ━━━━━━━━━━━━━━━━━━━━━━━━━━━━━━━━━━━━━━━━ 24.6/24.6 MB 8.7 MB/s eta 0:00:00
Collecting torchaudio===0.10.0+cu113
  Using cached https://download.pytorch.org/whl/cu113/torchaudio-0.10.0%2Bcu113-cp37-cp37m-linux_x86_64.whl
(2.9 MB)
Collecting typing-extensions
  Using cached typing_extensions-4.7.1-py3-none-any.whl (33 kB)
Requirement already satisfied: numpy in /home/ubuntu/miniconda3/envs/tl3.7/lib/python3.7/site-packages (from
 torchvision==0.11.1+cu113) (1.21.5)
Requirement already satisfied: pillow!=8.3.0,>=5.3.0 in /home/ubuntu/miniconda3/envs/tl3.7/lib/python3.7/sit
e-packages (from torchvision==0.11.1+cu113) (9.4.0)
Installing collected packages: typing-extensions, torch, torchvision, torchaudio
Successfully installed torch-1.10.0+cu113 torchaudio-0.10.0+cu113 torchvision-0.11.1+cu113 typing-extensions
-4.7.1
(tl3.7) ubuntu@magic:~/user_guide/transfer_learning/Modular_Latent_Space$
```

Then networkx is installed with:

```
pip install networkx==1.11
```

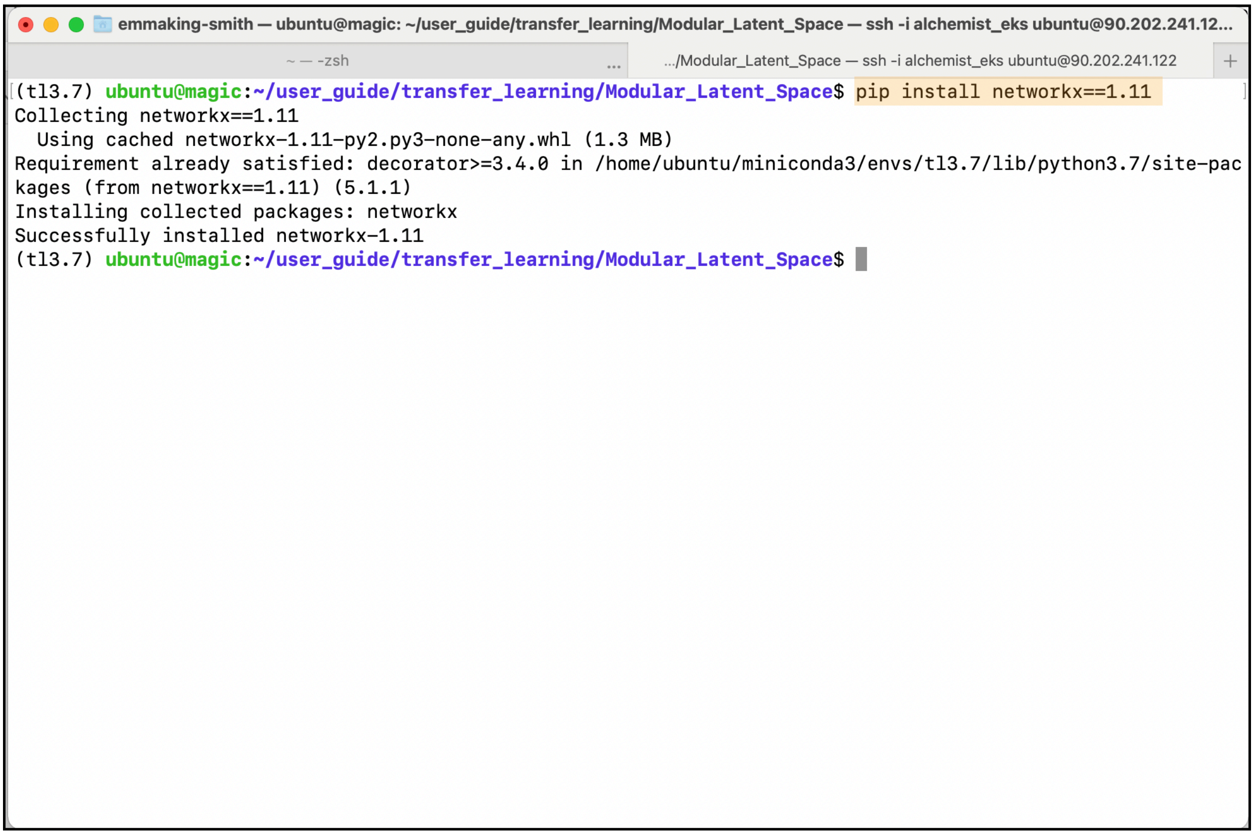

This is the most critical package to get the correct version. After version 1.11, the graph nomenclature changed and attempting to run the code with later versions will result in an error. **If you believe you are seeing a networkx error, please double check that the version you are running is 1.11.** This can easily be done using the following commands.

Start python - make sure you are in your virtual environment. Type:

```
python
```

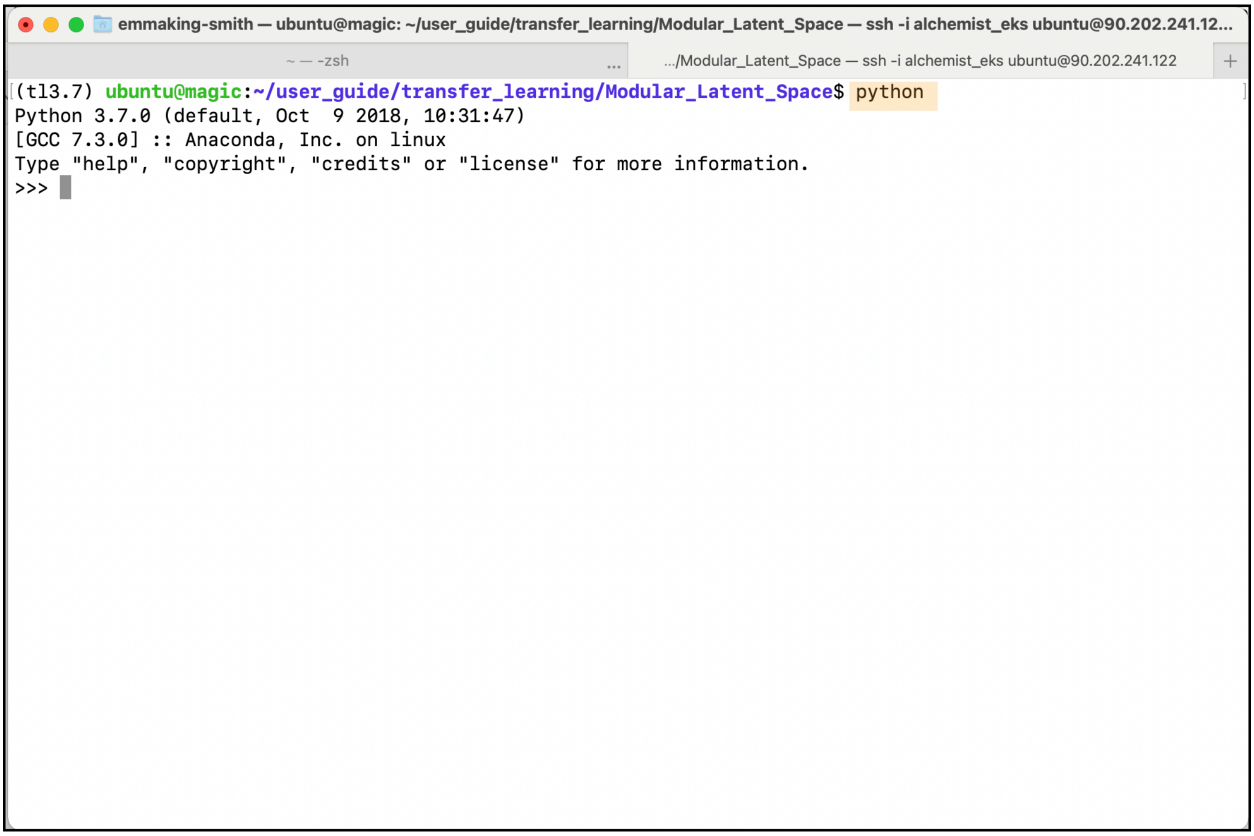

Import networkx with:

```
import networkx
```

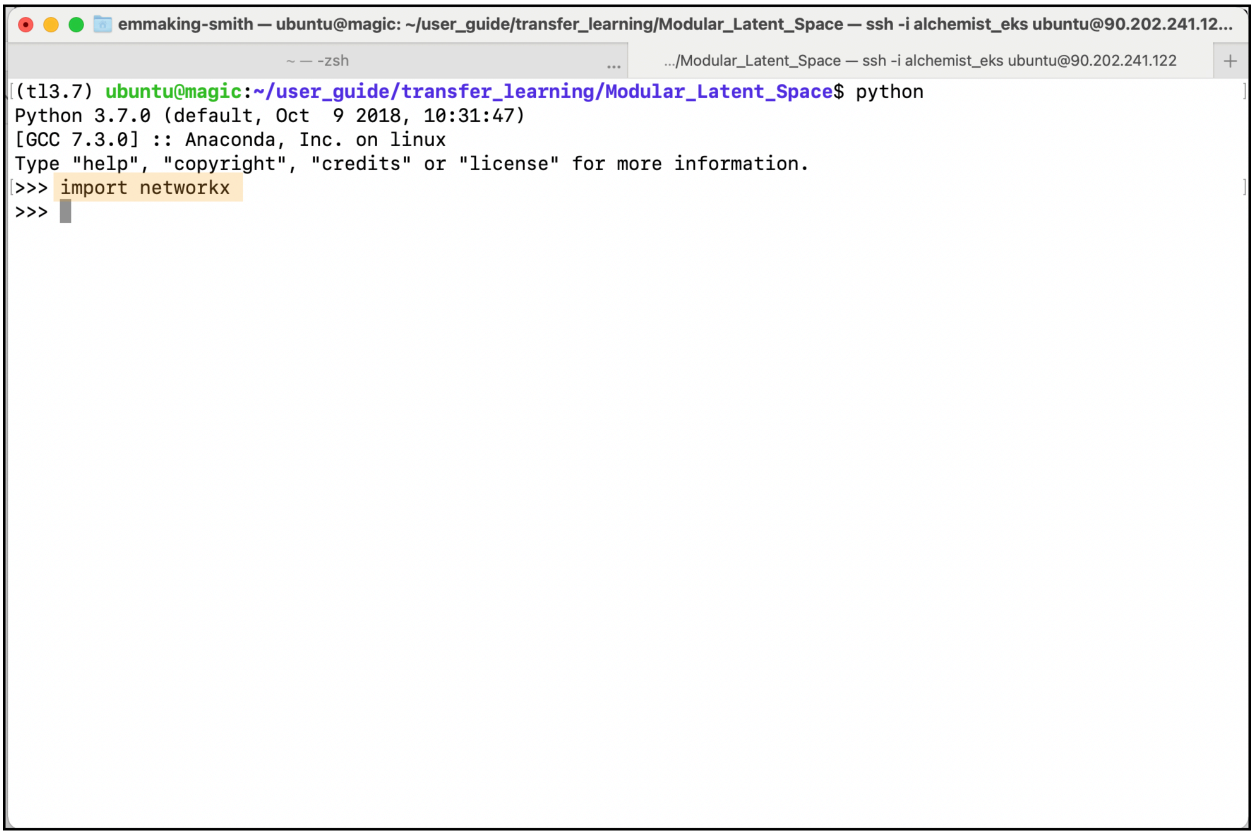

Check version with:

```
networkx.__version__
```

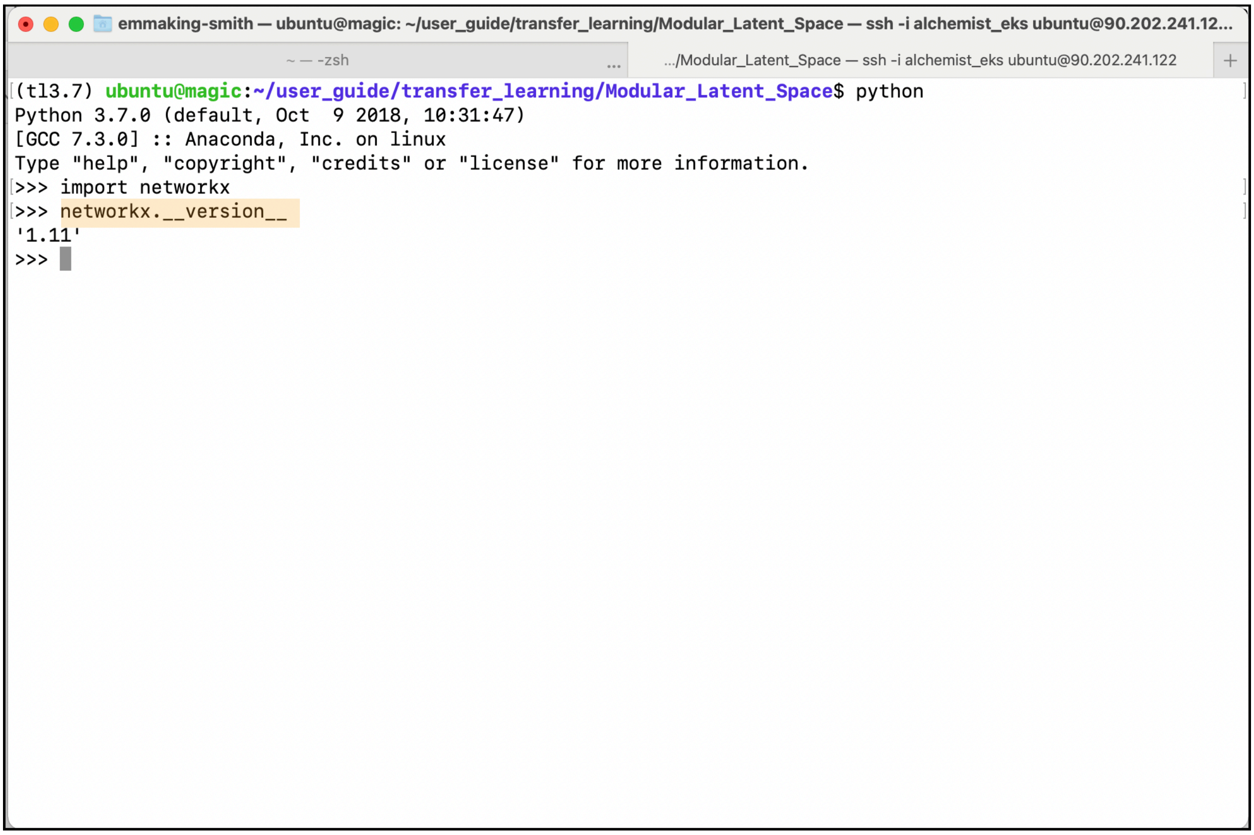

You should have an output of 1.11.

Next install numpy. This may have already been installed with a previous package.

```
pip install numpy==1.21.5
```

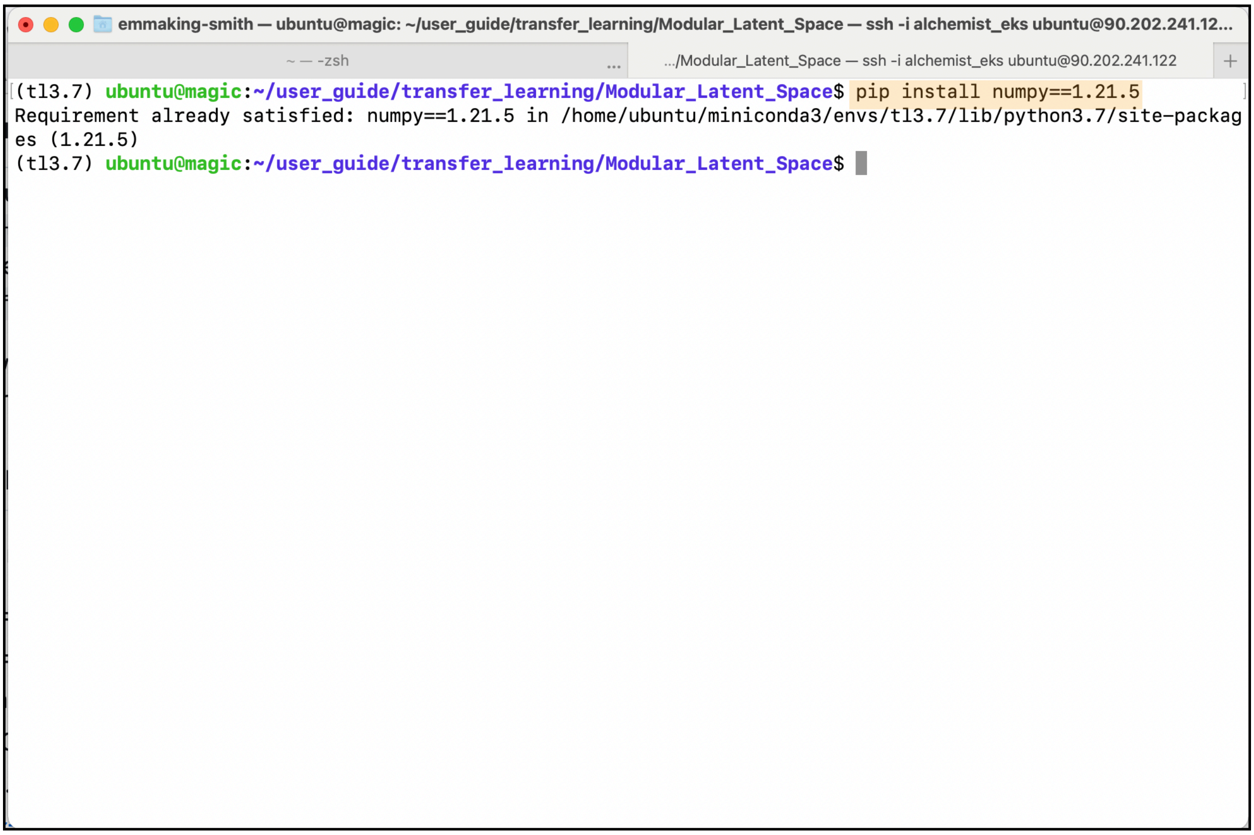

Then install pandas. Similar to numpy, this may already have been accomplished with a previous package installation.

```
pip install pandas==1.3.5
```

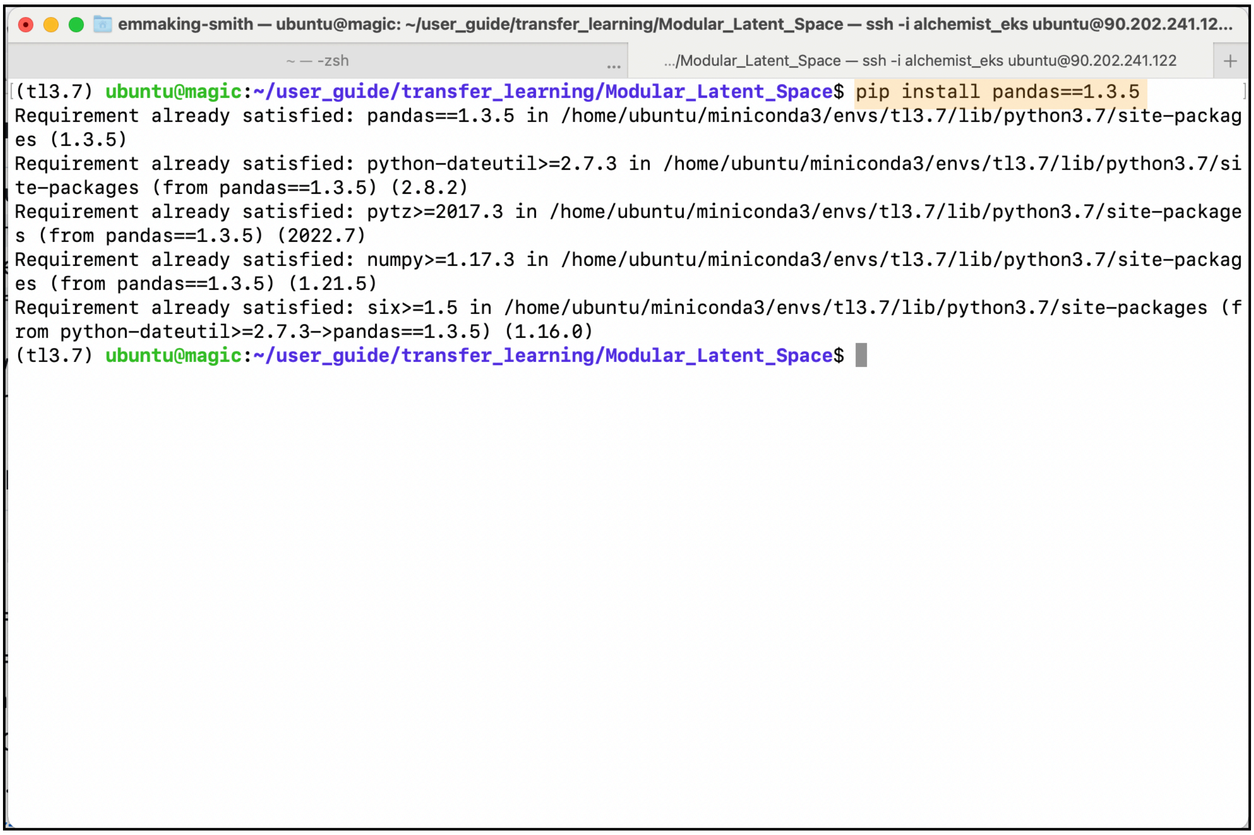

Finally, install sklearn.

```
pip install scikit-learn==1.0.2
```

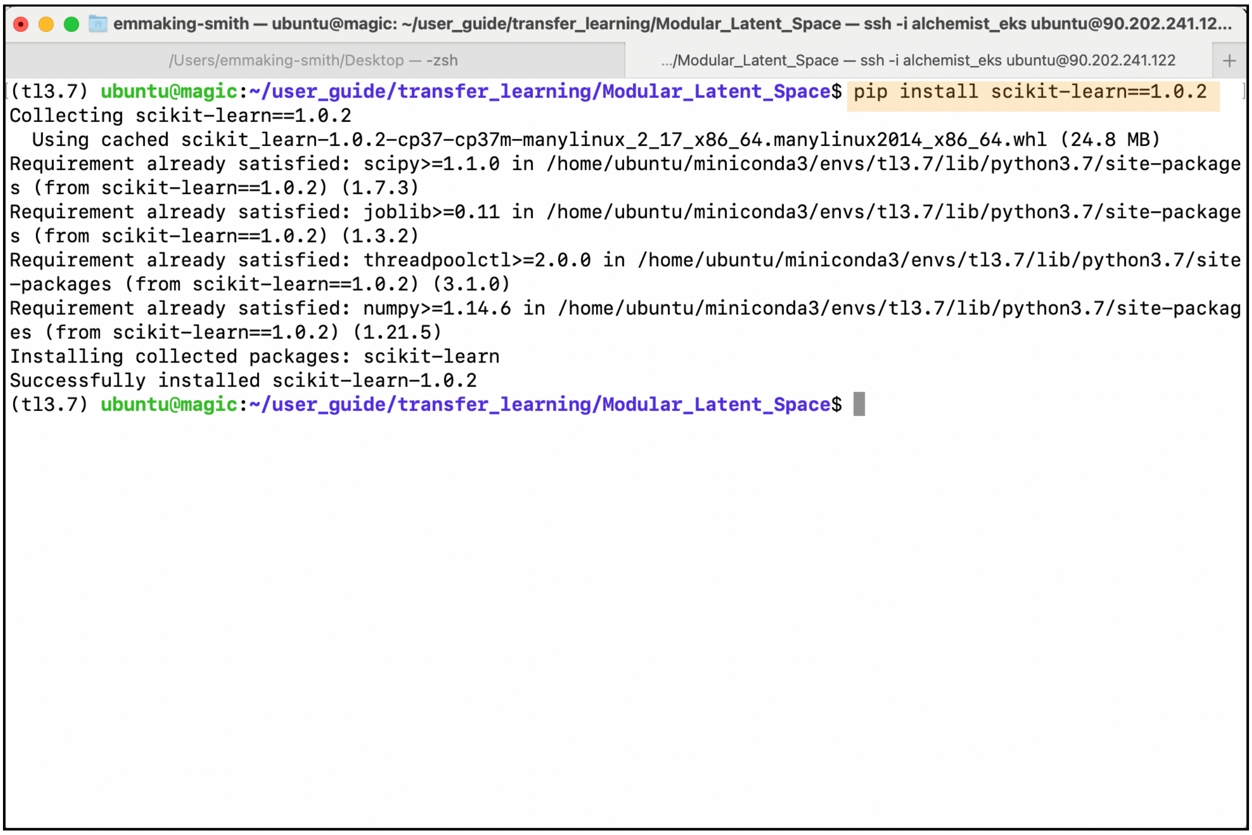

We are now all set up!

Run the Transfer Learning

We will be using the Buchwald-Hartwig dataset as our example transfer learning. Note that the pretrained layers from the crystal structure information will be frozen. This has been done for you and can be observed in Modular_Latent_Space/buchwald/buchwald_yield_mpnn lines 29-30 (see orange box in the figure below).

```python
device = 'cuda' if torch.cuda.is_available() else 'cpu'

class Buchwald_MPNN(nn.Module):
    def __init__(self, message_size, message_passes, atom_list, pretrained_mpnn_path):
        super(Buchwald_MPNN, self).__init__()

        self.message_size = message_size
        self.message_passes = message_passes
        self.atom_list = atom_list
        self.pretrained_mpnn_path = pretrained_mpnn_path

        self.mpnn = Big_MPNN(self.message_size, self.message_passes, self.atom_list)
        mpnn_trained_state_dict = self.gen_states(self.pretrained_mpnn_path)
        self.mpnn.load_state_dict(mpnn_trained_state_dict)

        # Turning off the params.
        for param in self.mpnn.parameters():
            param.requires_grad = False

        self.yield_predictor = nn.Sequential(
            nn.Linear(self.message_size * 4, self.message_size * 4),
            nn.ReLU(),
            # nn.Linear(self.message_size * 4, self.message_size * 4),
            # nn.ReLU(),
            # nn.Linear(self.message_size * 4, self.message_size * 4),
            # nn.ReLU(),
            # nn.Linear(self.message_size * 4, self.message_size * 4),
```

To run the transfer learning, we run a variation of following command:

```
python py_file_that_makes_predictions.py --option1 choice --option2 choice ...
```

The "--" indicates a flag. We are telling the model what arguments we are inputting. The flag lets the program know that a user-defined selection will occur, and the words after it detail the selection.

Understanding the Buchwald-Hartwig Flags:

For the Buchwald-Hartwig transfer learning, we have 3 flags of importance: split, test_mol_idx, and save_path. The split refers to what type of molecule should be left out for model validation. The options are "halide", "base", "ligand", and "additive". They are case sensitive.

The test_mol_idx stands for test molecule index and refers to which halide / base / ligand / additive molecules should be left out for model validation. Each index will yield a different set of molecules.

The final flag, save_path, is the place we wish to save our model and predictions. If we want to save it in a new directory called "predictions" to our Desktop our save_path flag would look like:

`--save_path ~/Desktop/predictions`.

To summarize our flags:

Flag name	Expected Value(s)	Example Flag Value	What to type
split	one of the following: halide,	ligand	`--split ligand`

	base, ligand, additive.<br><br>**NOTE:** Case sensitive!		
test_mol_idx	An integer between 0 and 3 if your split is **NOT** base.<br><br>An integer between 0 and 2 if your split **IS** base.	3*<br><br>*Must not have --split base flag.	`--test_mol_idx 3`
save_path	A path to a directory.	A new directory in the Modular_Latent_Space directory called transfer_learning_test.	`--save_path transfer_learning_test`<br><br>**NOTE:** Assuming your current location is the Modular_Latent_Space directory.

For more flags, please refer to Modular_Latent_Space/buchwald/buchwald_yield_prediction.py lines 25 - 37. For a basic transfer learning, feel free to use the default options.

```
19  from buchwald_yield_mpnn import Buchwald_MPNN
20
21  class Initialization:
22      def __init__(self):
23          pass
24
25      def init_args(self):
26          parser = argparse.ArgumentParser()
27          parser.add_argument('--path_to_buchwald_data', type=str, help='The path to the Buchwald csv file.',
28                              default='doyle_buchwald_data.csv')
29          parser.add_argument('--epochs', type=int, default=100)
30          parser.add_argument('--learning_rate', type=float, default=1e-4)
31          parser.add_argument('--batch_size', type=int, default=64)
32          parser.add_argument('--message_size', type=int, default=128)
33          parser.add_argument('--message_passes', type=int, default=3)
34          parser.add_argument('--pretrained_mpnn_path', type=str, default='../MPNN/big_mpnn_no_delocalised_no_unknown_model')
35          parser.add_argument('--save_path', '-s', type=str)
36          parser.add_argument('--split', type=str, help='Options are: halide, ligand, base, additive.')
37          parser.add_argument('--test_mol_idx', type=int, help='What molecule to leave out for testing.')
38          return parser.parse_args()
39
```

Run the Transfer Learning (Finally!):

We will then run the transfer learning on the Buchwald-Hartwig dataset. The module to do so is called buchwald_yield_prediction.py and is located in the buchwald directory. Move yourself into the buchwald directory using the `cd` command. If you are currently in the Modular_Latent_Space directory, this can be achieved with:

```
cd buchwald
```

You can easily tell you are now in the buchwald directory by looking at the path in blue. The final name will be "buchwald" (see orange box in figure below).

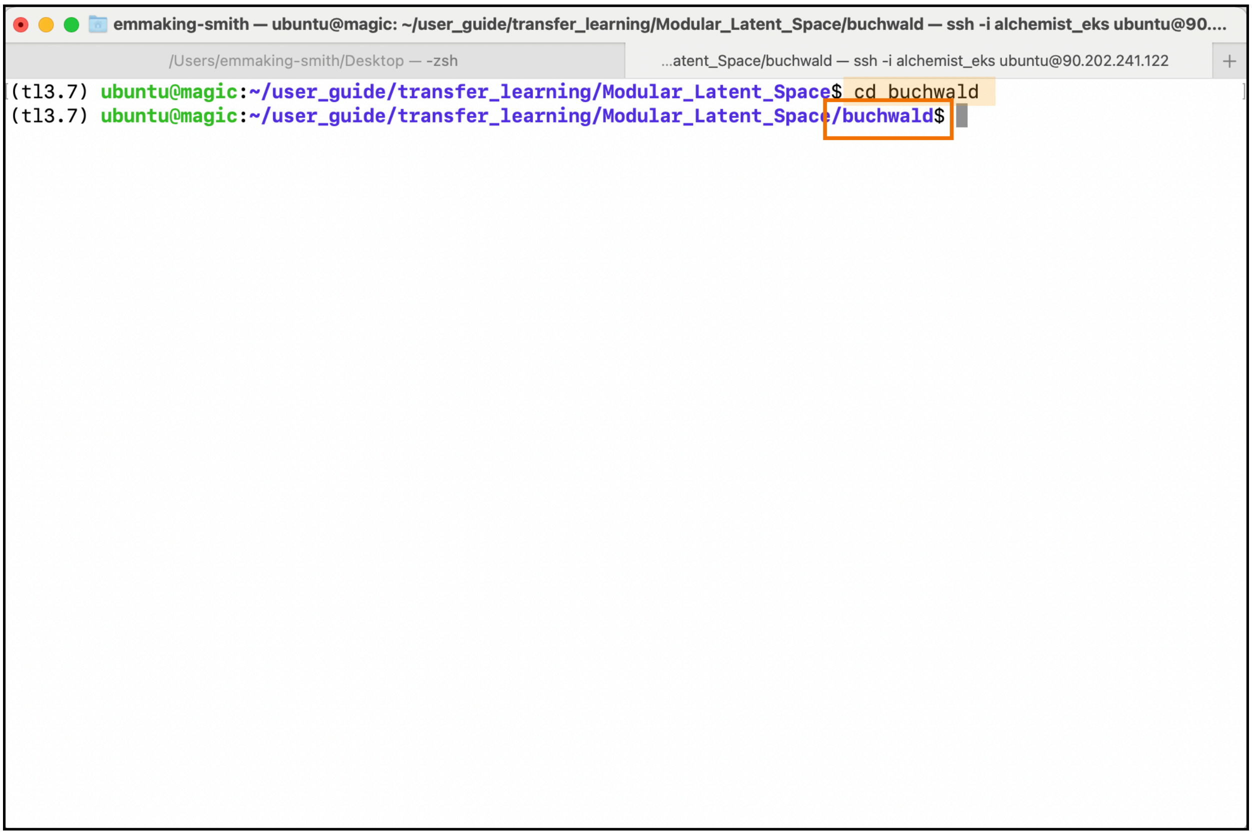

To run the Buchwald-Hartwig predictions, using a base split, with split index 2, and saving the predictions to a new directory called predictions/base_2, type:

```
python  buchwald_yield_prediction.py --split base --test_mol_idx 2 --
save_path predictions/base_2
```

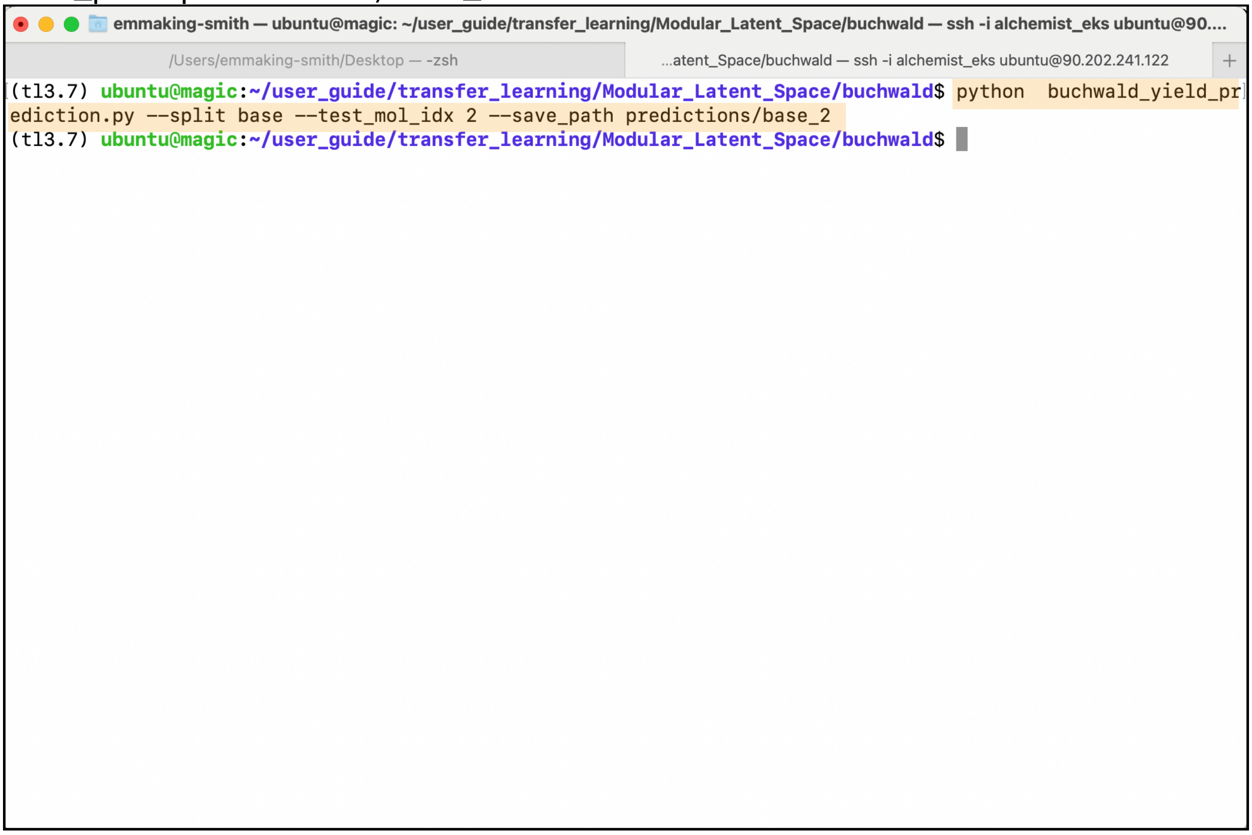

Helpful hint: If you are running these computations through an ssh, adding the nohup command to the beginning will keep your training running even if you lose the ssh connection:

```
nohup python  buchwald_yield_prediction.py --split base --test_mol_idx 2 --
save_path predictions/base_2
```

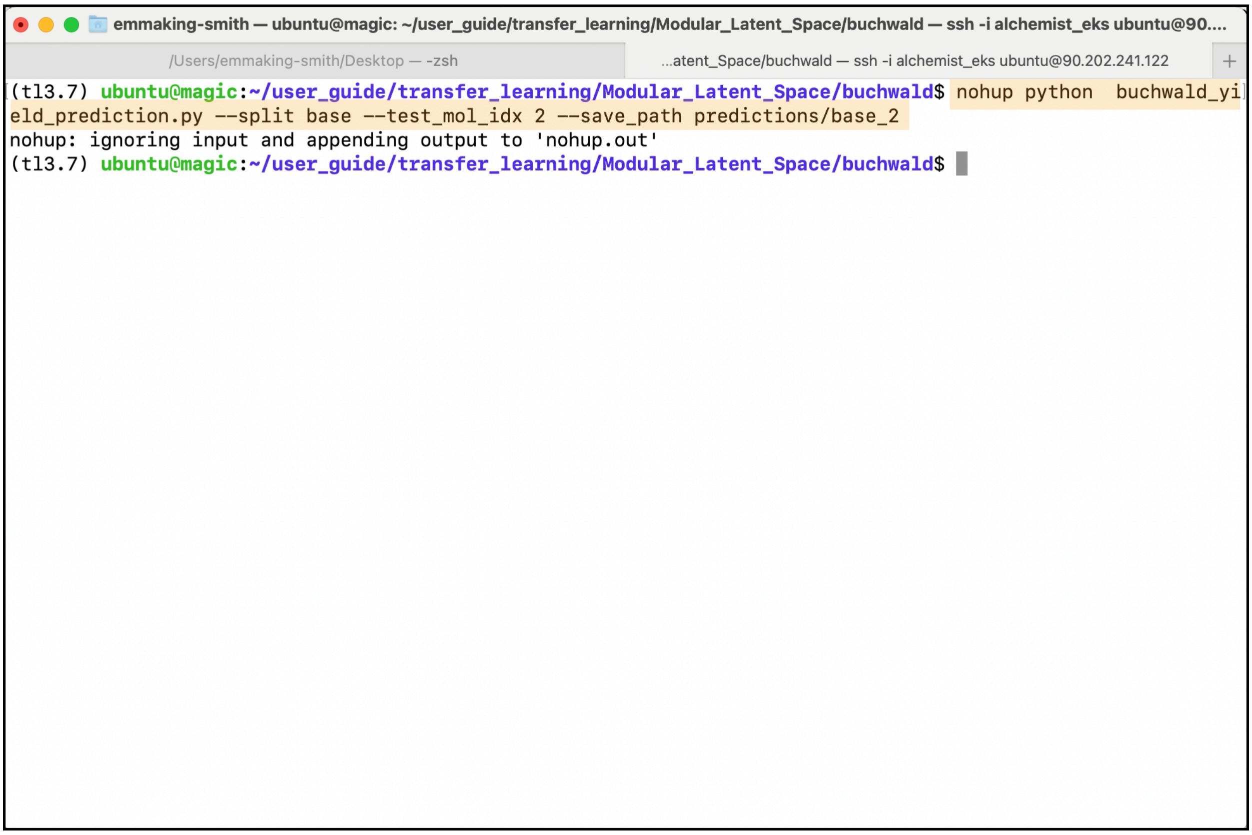

You can track your progress with the model_log.log (saved to the save_path directory) and the nohup output file (default nohup.out).

Voila! Your predictions will be saved to predictions/base_2.

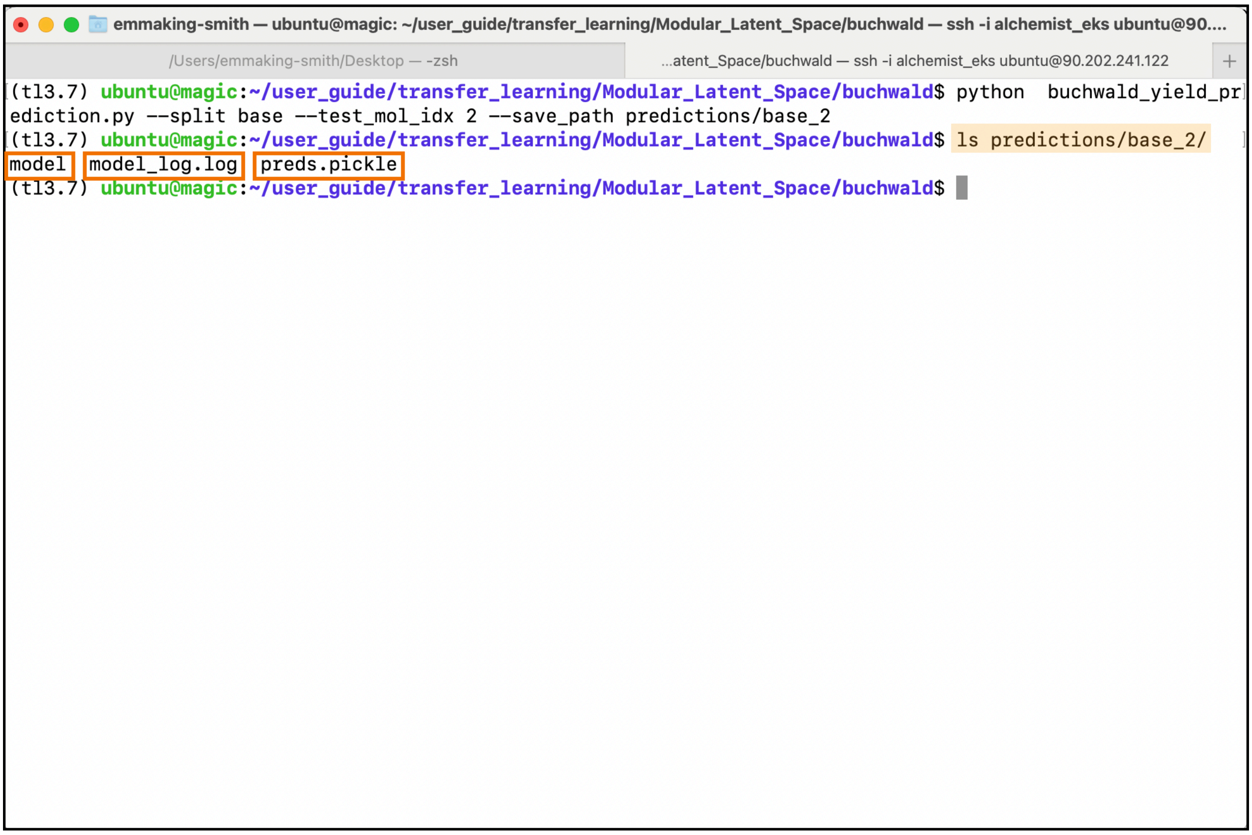

The preds.pickle are the predicted values, the model file is the final trained model, and the model_log.log is the log file that gives more information regarding the training loss and model parameters.

Viewing the Predictions

Ensure that you are in the correct directory (your save_path directory). If you are in the buchwald directory after completing training and have been following the naming convention of this document, you can move to the save_path directory by typing:

```
cd predictions/base_2/
```

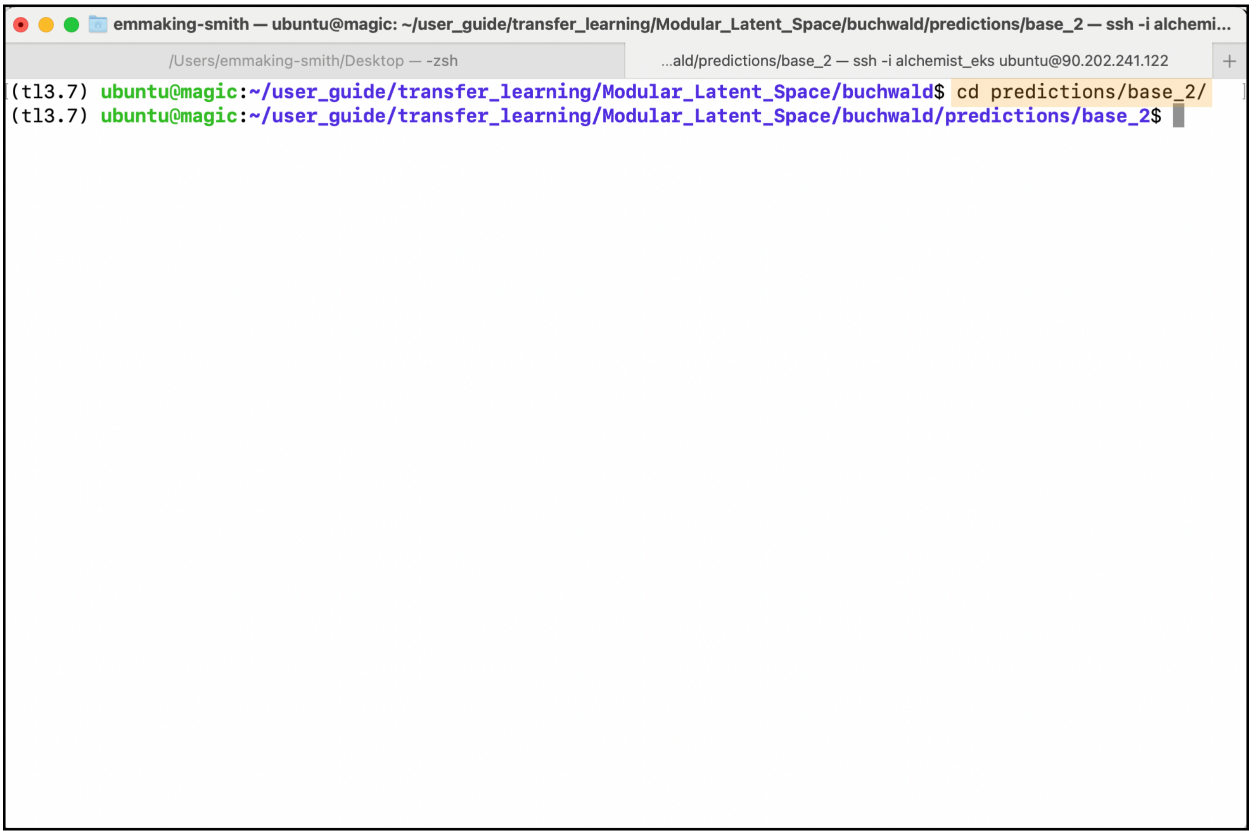

To view pickle files, go into python by typing:

```
python
```

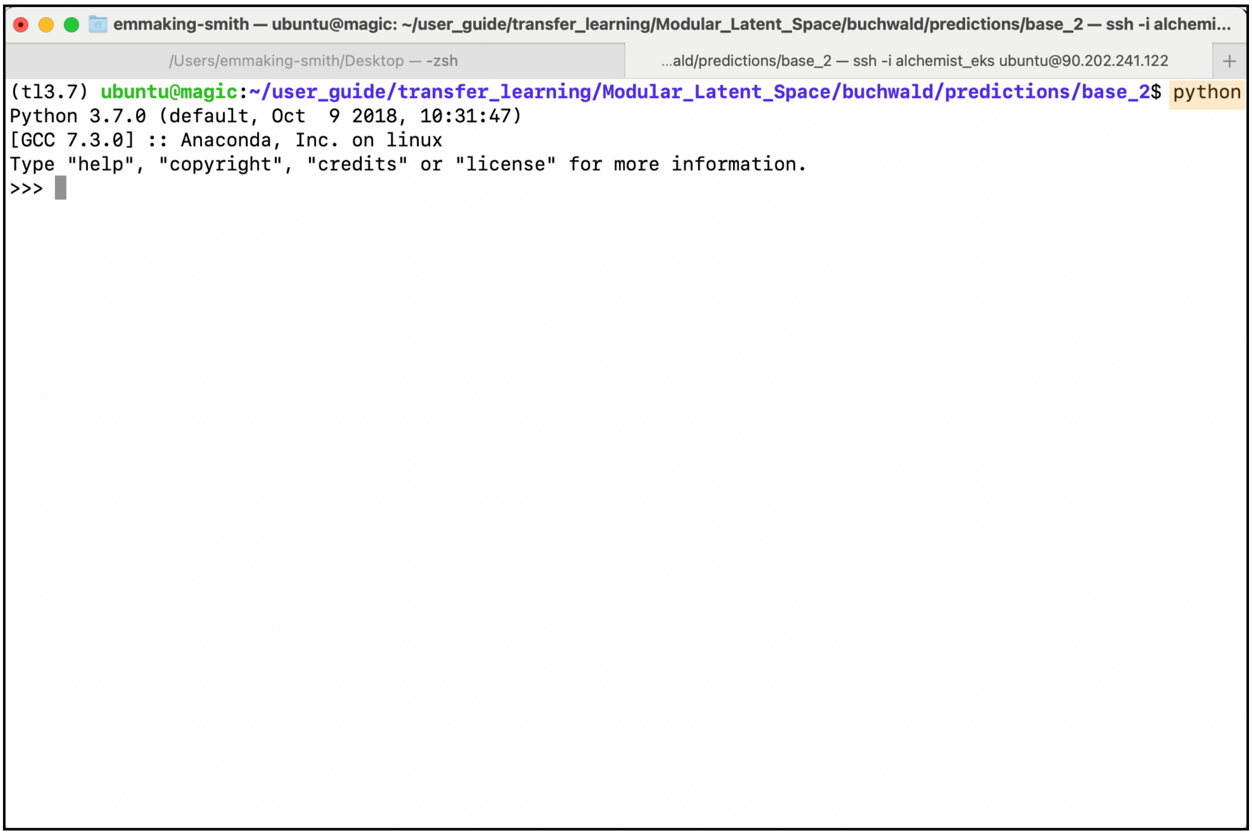

Import pandas with:

```
import pandas as pd
```

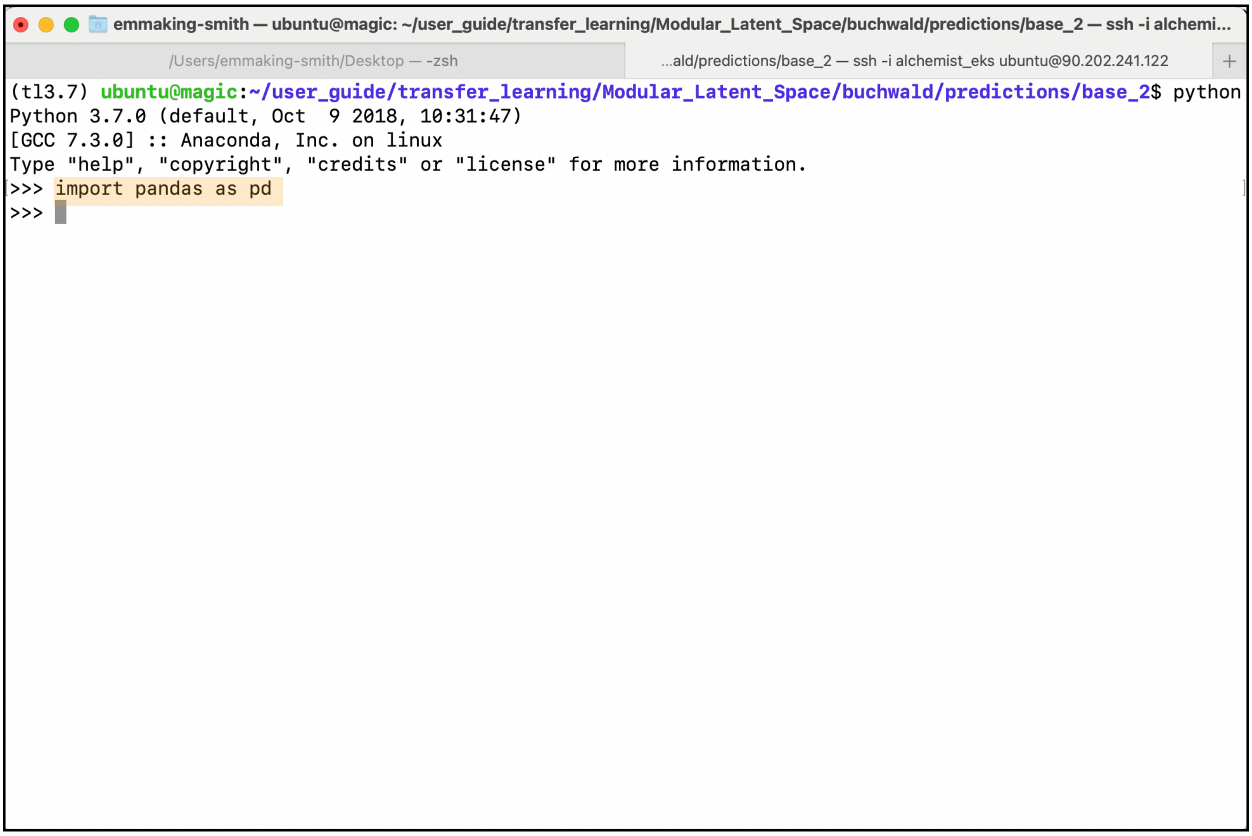

Import your predictions file with:

```
preds = pd.read_pickle('preds.pickle')
```

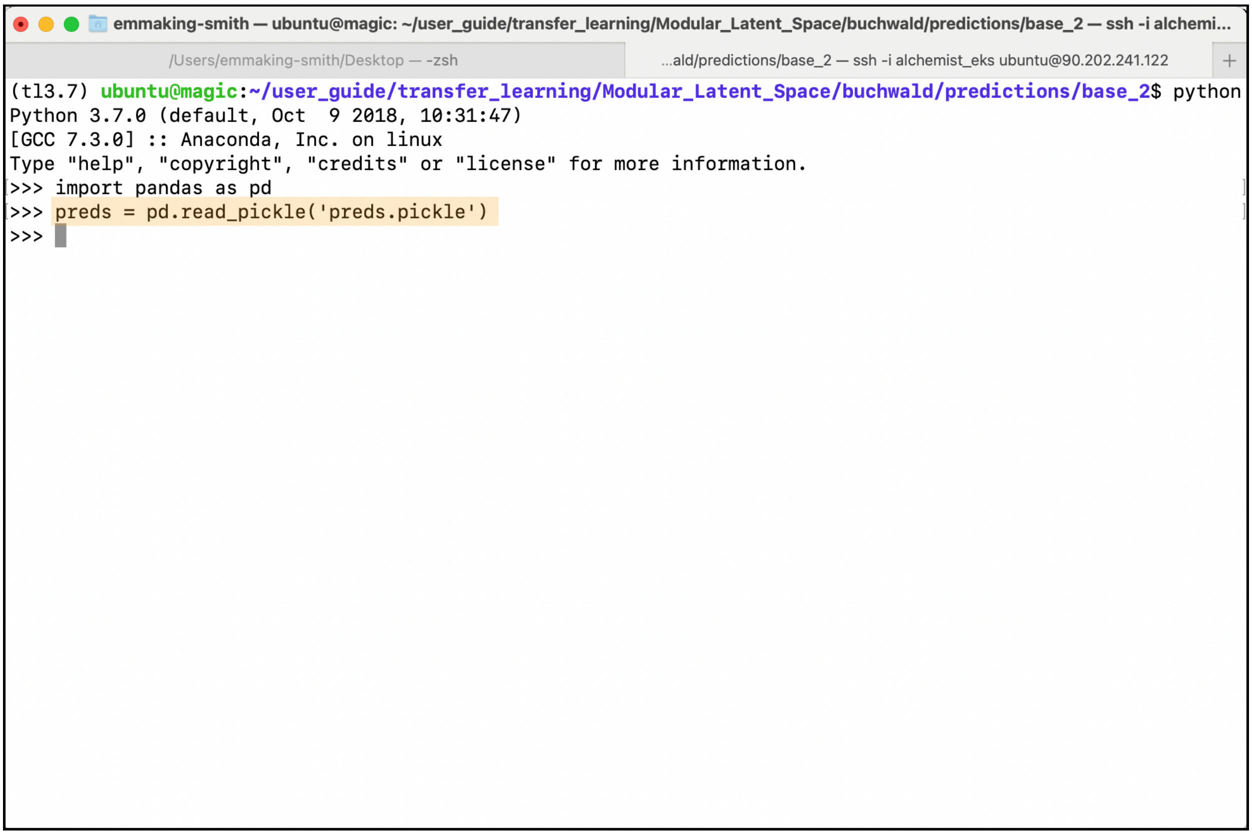

View your predictions file with:

```
preds
```

```
emmaking-smith — ubuntu@magic: ~/user_guide/transfer_learning/Modular_Latent_Space/buchwald/predictions/base_2 — ssh -i alchemi...
/Users/emmaking-smith/Desktop — -zsh        ...ald/predictions/base_2 — ssh -i alchemist_eks ubuntu@90.202.241.122
(tl3.7) ubuntu@magic:~/user_guide/transfer_learning/Modular_Latent_Space/buchwald/predictions/base_2$ python
Python 3.7.0 (default, Oct  9 2018, 10:31:47)
[GCC 7.3.0] :: Anaconda, Inc. on linux
Type "help", "copyright", "credits" or "license" for more information.
>>> import pandas as pd
>>> preds = pd.read_pickle('preds.pickle')
>>> preds
      plate  row  col  base  ...      yield  predicted_yield  true_yield       loss
0         1    1   17  BTMG  ...  20.671717        18.981762   20.671717   1.689955
1         1    1   18  BTMG  ...  37.065176        19.198864   37.065176  17.866312
2         1    1   19  BTMG  ...  40.108917        19.260603   40.108917  20.848314
3         1    1   20  BTMG  ...   1.571874        18.936052    1.571874  17.364179
4         1    1   21  BTMG  ...  13.579588        19.195978   13.579588   5.616390
...     ...  ...  ...   ...  ...        ...              ...         ...        ...
1524      3   32   28  BTMG  ...  54.862777        25.901226   54.862777  28.961551
1525      3   32   29  BTMG  ...   1.720185        25.410936    1.720185  23.690751
1526      3   32   30  BTMG  ...  52.962783        25.581123   52.962783  27.381660
1527      3   32   31  BTMG  ...  55.264663        25.629990   55.264663  29.634674
1528      3   32   32  BTMG  ...   0.000000        17.102970    0.000000  17.102970

[1529 rows x 20 columns]
>>>
```

This will give you a snapshot of the prediction file. You can save it out as a csv file which is openable with Microsoft Excel if that is more convenient for you with:

```
preds.to_csv('preds.csv')
```

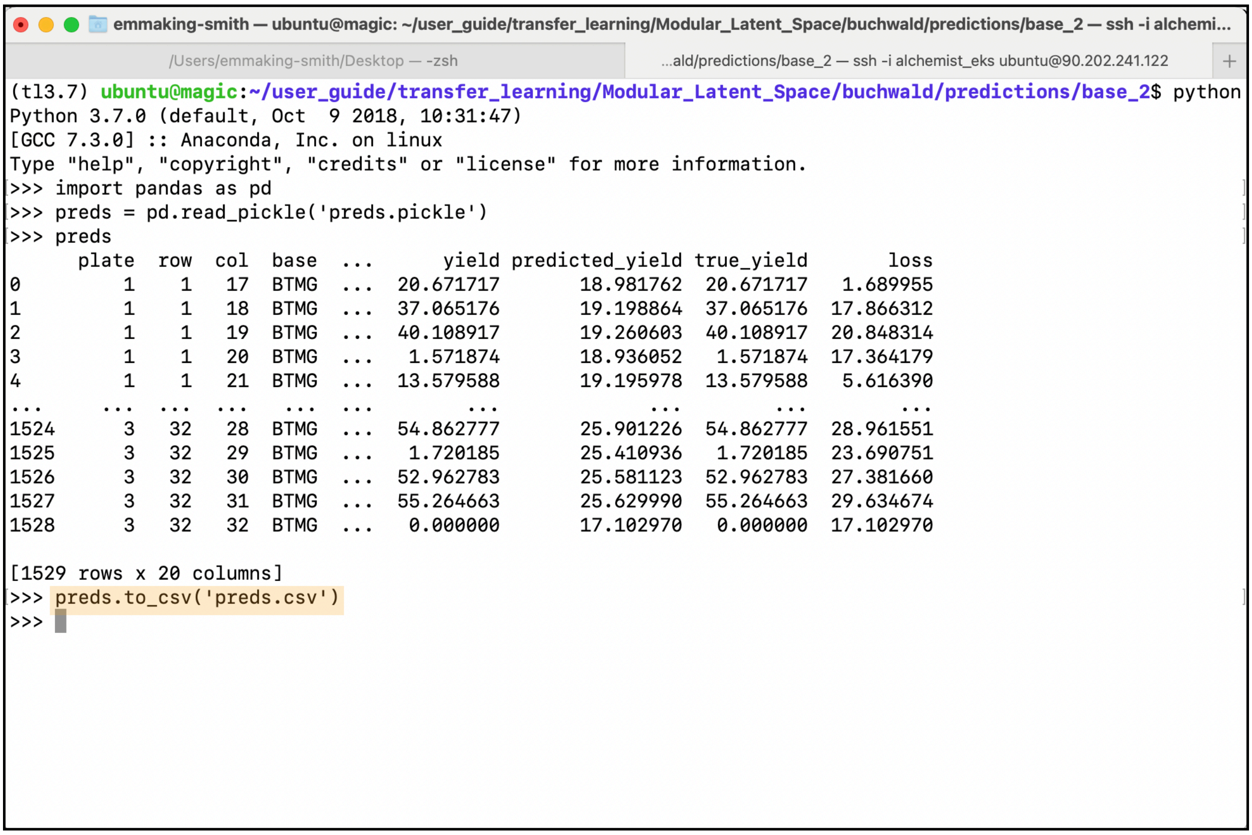


```
(tl3.7) ubuntu@magic:~/user_guide/transfer_learning/Modular_Latent_Space/buchwald/predictions/base_2$ python
Python 3.7.0 (default, Oct  9 2018, 10:31:47)
[GCC 7.3.0] :: Anaconda, Inc. on linux
Type "help", "copyright", "credits" or "license" for more information.
>>> import pandas as pd
>>> preds = pd.read_pickle('preds.pickle')
>>> preds
      plate  row  col  base  ...      yield  predicted_yield  true_yield       loss
0         1    1   17  BTMG  ...  20.671717        18.981762   20.671717   1.689955
1         1    1   18  BTMG  ...  37.065176        19.198864   37.065176  17.866312
2         1    1   19  BTMG  ...  40.108917        19.260603   40.108917  20.848314
3         1    1   20  BTMG  ...   1.571874        18.936052    1.571874  17.364179
4         1    1   21  BTMG  ...  13.579588        19.195978   13.579588   5.616390
...     ...  ...  ...   ...  ...        ...              ...         ...        ...
1524      3   32   28  BTMG  ...  54.862777        25.901226   54.862777  28.961551
1525      3   32   29  BTMG  ...   1.720185        25.410936    1.720185  23.690751
1526      3   32   30  BTMG  ...  52.962783        25.581123   52.962783  27.381660
1527      3   32   31  BTMG  ...  55.264663        25.629990   55.264663  29.634674
1528      3   32   32  BTMG  ...   0.000000        17.102970    0.000000  17.102970

[1529 rows x 20 columns]
>>> preds.to_csv('preds.csv')
>>>
```

This will generate a csv file in your working directory called preds.

Thank you for reading to the end of this guide!

We hope it has been a helpful resource for you.
